# Synthesis, effect of substituents on the regiochemistry and equilibrium studies of tetrazolo[1,5-*a*]pyrimidine/2-azidopyrimidines

**DOI:** 10.3762/bjoc.13.237

**Published:** 2017-11-10

**Authors:** Elisandra Scapin, Paulo R S Salbego, Caroline R Bender, Alexandre R Meyer, Anderson B Pagliari, Tainára Orlando, Geórgia C Zimmer, Clarissa P Frizzo, Helio G Bonacorso, Nilo Zanatta, Marcos A P Martins

**Affiliations:** 1 Laboratório de Química, Universidade Federal do Tocantins, Palmas, TO 77001-090, Brazil; 2 Núcleo de Química de Heterociclos (NUQUIMHE), Department of Chemistry, Federal University of Santa Maria (UFSM), 97105-900, Santa Maria, RS, Brazil

**Keywords:** 5-aminotetrazol, azide–tetrazole equilibrium, 2-azidopyrimidine, β-enaminones, tetrazolo[1,5-*a*]pyrimidine, trifluoromethylatedtetrazolo[1,5-*a*]pyrimidines

## Abstract

An efficient synthesis methodology for a series of tetrazolo[1,5-*a*]pyrimidines substituted at the 5- and 7-positions from the cyclocondensation reaction [CCC + NCN] was developed. The NCN corresponds to 5-aminotetrazole and CCC to β-enaminone. Two distinct products were observed in accordance with the β-enaminone substituent. When observed in solution, the compounds can be divided into two groups: (a) precursor compounds with R = CF_3_ or CCl_3_, which leads to tetrazolo[1,5-*a*]pyrimidines in high regioselectivity with R at the 7-position of the heterocyclic ring; and (b) precursor compounds with R = aryl or methyl, which leads to a mixture of compounds, tetrazolo[1,5-*a*] pyrimidines (R in the 5-position of the ring) and 2-azidopyrimidines (R in the 4-position of the ring), which was attributed to an equilibrium of azide–tetrazole. In the solid state, all compounds were found as 2-azidopyrimidines. The regiochemistry of the reaction and the stability of the products are discussed on the basis of the data obtained by density functional theory (DFT) for energetic and molecular orbital (MO) calculations.

## Introduction

Tetrazolo[1,5-*a*]pyrimidines have attracted attention in the pharmaceutical field due to their significant potential to exhibit antitumor, antimicrobial, and antioxidant activities [1–2]. In addition to acting as a stimulant of the central nervous system [3], this class of substances plays an important role in drugs that are used to treat obesity, diabetes, hypertension, coronary heart disease, thyroid cancer, and hepatitis B virus [4–13].

Although many synthetic procedures used to obtain azolopyrimidines have been described in the literature [1,14–16], the synthesis of tetrazolo[1,5-*a*]pyrimidines, especially those that are formed from 5-aminotetrazole, such as 1,3-binucleophile synthons, have not been sufficiently discussed [17]. Today, several existing methods are known to have several disadvantages, including long reaction time, the use of harmful solvents, and multistep synthesis [18–19].

Moreover, researchers in the 1960s and 1970s reported the existence of an azide–tetrazole equilibrium in many heterocyclic systems, namely tetrazolopyridines, tetrazolopyridazines, tetrazolopyrimidines, tetrazoloazines, and tetrazolopurines. Both tetrazole and azide have different chemical properties. Among other factors, the nature of the substituents, solvent, temperature, and physical state of the compound (solid state or solution) are the properties that have the most influence on the azide–tetrazole equilibrium [20–28].

A comprehensive understanding of the azide–tetrazole equilibrium is of great interest from a pharmacological point of view. A broader control and modulation of the equilibrium can lead to a specific compound to prevent undesirable pharmacokinetics and biological properties arising from the differences in the chemical structure.

Our research group previously demonstrated an efficient and regioselective synthesis of pyrazolo[1,5-*a*]pyrimidines and aryl[heteroaryl]pyrazolo[1,5-*a*]pyrimidines in acetic acid under reflux. The regioselectivity was attributed to the high nucleophilicity of the amino group in 3-amino-5-methyl-1*H*-pyrazole and the high electrophilicity of the β-carbon atom of the enone, both soft sites of the starting materials [29]. In recent research, we developed an efficient method to obtain 1,2,4-triazolo[1,5-*a*]pyrimidines from the cyclocondensation reaction of 1,1,1-trifluoro-4-metoxy-3-alken-2-one or β-enaminones with 5-amino-1,2,4-triazole. The methodology using ultrasound irradiation promoted shorter reaction times, high regioselectivity, and excellent yields, when compared with conventional thermal heating [30]. Presently, we demonstrate an eco-friendly synthesis of 5- and 7-substituted tetrazolo[1,5-*a*]pyrimidine isomers, in good to excellent yields, using ionic liquids and water as solvents, and short reaction times. In addition, 5-substituted isomers have proved to be excellent models in carrying out a thorough investigation of the azide–tetrazole equilibrium mechanism by density functional theory (DFT) and various experimental methods (e.g., NMR, SCXRD, PXRD, FTIR).

Therefore, this study aims to (i) propose an efficient methodology for the synthesis of tetrazolo[1,5-*a*]pyrimidines substituted at the 5- and 7-positions of the heterocyclic ring; (ii) observe the regiochemistry of the cyclocondensation reaction and the effect of the substituent on the β-enaminone precursor and (iii) elucidate the azide–tetrazole equilibrium when the compound is in solid form or dissolved in distinct solvents. The tetrazolo[1,5-*a*]pyrimidine synthesis was conducted from a cyclocondensation reaction of the type [CCC + NCN], in which the CCC block is a series of β-dimethylamino vinyl ketones, and the NCN block is 5-aminotetrazole.

## Results and Discussion

### Synthesis of tetrazolo[1,5-*a*]pyrimidines

The β-dimethylamino vinyl ketones (β-enaminones) were synthesized using methodologies previously described by our research group [31]. In order to achieve better reaction conditions for the preparation of tetrazolo[1,5-*a*]pyrimidines, the reaction of β-enaminone **1a** with 5-aminotetrazole (**2**) was carried out under several conditions using conventional solvents and ionic liquids (ILs) to provide 5-phenyltetrazolo[1,5-*a*]pyrimidine (**3a**).

Two conditions in which the product exceeded 80% yield were found: (i) toluene reflux in the presence of HCl for 16 h (entry 2, Table 1), and (ii) ionic liquid [HMIM][TsO] at 120 °C in the presence of HCl for 5 min or 2.5 h (entries 7 and 8, respectively, Table 1). Nevertheless, the use of the IL [HMIM][TsO] proved advantageous when compared to toluene due to the considerable difference in reaction time (IL (5 min) in comparison with toluene (16 h)). This can be attributed to the significant stabilization of charges in the activated complex of the reaction promoted by charged [HMIM][TsO]. Other advantages regarding special properties (such as high thermal stability and low volatility) of ILs can be considered. Reactions in [BMIM][BF_4_] and [HMIM][TsO] using *p*-toluenesulfonic acid (*p*-TsO) as a catalyst were unsuccessful and have been omitted from Table 1.

**Table 1:** Reaction conditions for the synthesis of compounds **3a**/**4a**.

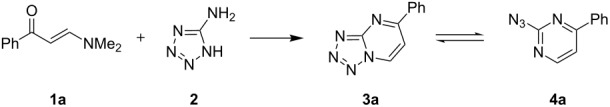					

entry	solvent^a^	temp. (°C)	time	acid^b^	yield (%)^c^

1	AcOH	reflux	16 h	–	0
2	toluene	reflux	16 h	HCl	83
3	[BMIM][BF_4_]	120	6 h	–	0
4	[BMIM][BF_4_]	120	5 min	HCl	0
5	[BMIM][BF_4_]	120	2.5 h	HCl	54
6	[BMIM][BF_4_]	120	6 h	HCl	60
7	[HMIM][TsO]	120	5 min	HCl	82
8	[HMIM][TsO]	120	2.5 h	HCl	80
9	[HMIM][TsO]	120	6 h	HCl	63

^a^Solvent: 5 mL; IL: 1 mmol. ^b^0.1% of catalyst. ^c^Isolated product yield.

The best conditions that were found for **1a** and 5-aminotetrazole (**2**) were used for the reaction of **2** with β-enaminones **1b**–**i** (Table 2). The products formed from the reaction of **1a–g** with 5-aminotetrazole were detected as a tetrazolo[1,5-*a*]pyrimidine
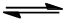
2-azidopyrimidine (**3**
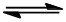
**4**) equilibrium in solution, which were identified by NMR. On the other hand, the reaction of **1h**,**i** with 5-aminotetrazole (**2**) was highly regioselective and yielded tetrazolo[1,5-*a*]pyrimidines **5h**,**i**, in which R = CF_3_ and CCl_3_ and presented exclusively R at the 7-position of the heterocyclic ring (Table 2). The tetrazolo[1,5-*a*]pyrimidine
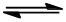
2-azidopyrimidine (**3**
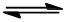
**4**) equilibrium will be discussed in detail later in this article. All products were formed in good to excellent yields (40–96%). To the best of our knowledge, all synthesized compounds, with the exception of **3a**:**4a**, **3g**:**4g**, and **5h** [21,32], have not been previously described in the literature. Acid catalysts and conventional organic solvents were used for the cyclization of enaminones [33–34]. This method provided good yields and regioselectivity using a simple catalyst and an IL as the solvent. Notably, not only the reaction time was short, but the purification process is simple.

**Table 2:** Synthesis of compounds **3a–g**, **4a–g** and **5h**,**i**.

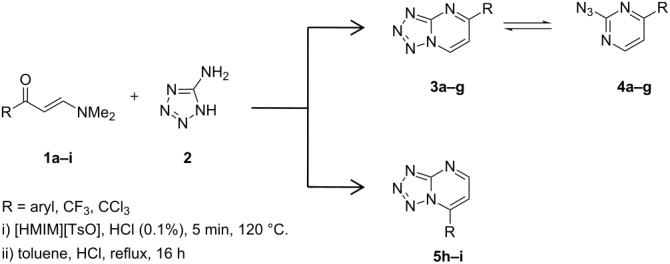				

product	R	yield^a^ (%)		molar ratio^b^
		[HMIM][TsO]	toluene	**3**:**4**

**3a**:**4a**	Ph	82	87	84:16
**3b**:**4b**	4-F-C_6_H_4_	83	85	79:21
**3c**:**4c**	4-Cl-C_6_H_4_	91	88	85:15
**3d**:**4d**	4-Br-C_6_H_4_	95	87	79:21
**3e**:**4e**	4-I-C_6_H_4_	86	96	89:11
**3f**:**4f**	4-CH_3_-C_6_H_4_	89	91	93:7
**3g**:**4g**	4-OCH_3_-C_6_H_4_	93	78	94:6
**5h**	CF_3_	70	40	100^c^
**5i**	CCl_3_	68	77	100^c^

^a^Isolated product yield. ^b^The **3**:**4** molar ratio was calculated based on the aromatic hydrogens in the ^1^H NMR spectrum using DMSO-*d*_6_ for products obtained in [HMIM][TsO]. Compounds **3a**:**4a**, **3g**:**4g** were already published [21] in a proportion of 59:41 and 57:43 in CDCl_3_, respectively. Compound **5h** was previously observed as 1,7-isomer [32]. ^c^Was only observed as a single product in CDCl_3_.

### The regiochemistry of tetrazolo[1,5-*a*]pyrimidines

**Figure 1:**
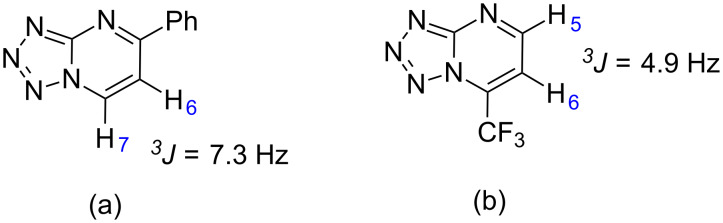
Hydrogen coupling constants (^3^*J*_H-H_) of (a) H6–H7 for **3a** and (b) H5–H6 for **5h**.

The regiochemistry of tetrazolo[1,5-*a*]pyrimidines can be verified by ^1^H NMR spectroscopy. The substituted tetrazolo[1,5-*a*]pyrimidines found in the literature [21,32] presented coupling constants of *^3^J*_H-H =_ 6.9–7.3 Hz for H6–H7, and *^3^J*_H-H_ = 4.2–4.8 Hz for H5–H6. The coupling constant for compound **3a** is *^3^J*_H-H_ = 7.3 Hz, while compound **5h** has a coupling constant of *^3^J*_H-H_ = 4.9 Hz, which characterizes the substitution at the 5-position and 7-position of the heterocyclic ring, respectively (Figure 1).

The formation of 7-trihalomethyl-substituted (R = CF_3_ and CCl_3_) tetrazolo[1,5-*a*]pyrimidine when β-enaminones were reacted with 5-aminotetrazole showed that the structure of the R group affects the regiochemistry of the products formed.

The β-enaminones **1a**–**g** (R = aryl) result predominantly in 5-aryl-substituted tetrazolo[1,5-*a*]pyrimidines **3a**–**g**. β-Enaminones **1h**,**i** (R = CF_3_ and CCl_3_) result in a highly regioselective synthesis of tetrazolo[1,5-*a*]pyrimidines **3h**,**i** with CX_3_ groups at the 7-position of the fused pyrimidine ring.

**Figure 2:**
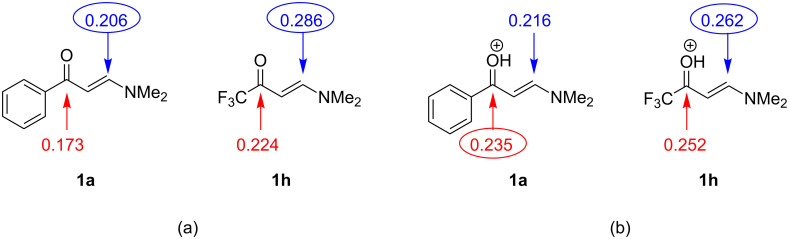
LUMO coefficients for (a) β-enaminones **1a**,**h**, and their (b) conjugated acids.

The well-defined regiochemistry of these reactions, according to the substituent, can be understood by determination of the LUMO coefficients of the carbonyl (C1) and the β-carbon (C3) of the β-enaminones. The LUMO coefficient values for neutral and conjugated acids of β-enaminones with R = phenyl (**1a**) and CF_3_ (**1h**) were determined using the Gaussian 09 software package [35] and calculated at B3LYP/cc-pVTZ level of theory (Figure 2).

Under neutral conditions, the highest LUMO coefficient in the β-enaminones with R = phenyl and CF_3_ can be found at the β-carbon C3 (Figure 2a). Under acidic conditions, in which the reactions were performed, C1 is the carbon with the highest LUMO coefficient (0.235) when R = phenyl (Figure 2b). Consequently, the first nucleophilic attack from the amino group of 5-aminotetrazole occurs at C1, leading to the formation of the 5-aryl-substituted compound **3a**. On the other hand, when R = CF_3_, the highest LUMO coefficient (0.262) is located at the C3 carbon of the β-enaminone (Figure 2b). Therefore, the initial nucleophilic attack leads to the formation of the 7-trifluoromethyl-substituted compound **5h**. The first nucleophilic attack from the amino group of 5-aminotetrazole was evidenced by HOMO coefficient calculations (Table S3 in Supporting Information File 1). The highest HOMO coefficient for the NH_2_ group indicates the superior nucleophilicity of the NH_2_ group in the aminotetrazole molecule. A scheme that illustrates the first nucleophilic attack that leads to different isomers according to the β-enaminone substituent can be seen in Figure S1 in Supporting Information File 1.

The synthesis of tetrazolo[1,5-*a*]pyrimidines **3a**–**g** and 2-azidopyrimidines **4a**–**g** were confirmed from the ^1^H and ^13^C NMR spectra and mass spectrometry. Chemical shift assignments and coupling constants of pyrimidine ring hydrogens were compared with compounds **3a**, **4a**, **3g**, **4g**, and **5h** described in the literature [21,32]. The HMQC and HMBC ^1^H–^13^C were carried out in order to confirm the signal attributions (see Figures S5 and S6 in Supporting Information File 1).

The ^1^H NMR spectra of **3a**–**g**:**4a**–**g** show the aromatic protons H6 and H7 as doublets in the region between 8.11–8.24 ppm and 9.69–9.82 ppm, respectively, with ^3^*J*_H-H_ = 7.3 Hz attributed to 5-aryl-substituted tetrazolo[1,5-*a*]pyrimidines. The **4a–g** signals show the H5 and H6 aromatic hydrogens as doublets at 7.78–7.89 ppm and 8.67–8.79 ppm, respectively, where ^3^*J*_H-H_ = 5.3 Hz characterizes the azide derivatives. The analysis of the ^1^H NMR data of compounds **5h**,**i** shows the aromatic protons H5 and H6 as doublets in the region between 7.38–7.67 ppm and 8.80–8.85 ppm, respectively. The ^3^*J*_H-H_ coupling constants of the aromatic H5 and H6 protons are around 4.8–5.2 Hz, which is a characteristic of the 7-substituted compound.

The ^13^C NMR spectra of **3a**–**g**:**4a**–**g** show C6 from δ 109.7 to 110.9, C7 from δ 135.1 to 143.3, C3a from δ 154.7 to 155.3, and the carbon C5 from δ 163.4 to 165.0, respectively. The ^13^C NMR data for **4a**–**g** show C5 from δ 112.7 to 113.5, C6 from δ 135.5 to 160.9, C2 from δ 160.3 to 163.9, and C4 from δ 163.0 to 167.4, respectively. The analysis of the ^13^C NMR data of compounds **5h**,**i** shows C6 from δ 111.3 to 112.3, C7 from δ 157.7 to 161.5, C3a from δ 161.5 to 162.3, and C5 from δ 163.2 to 168.0, respectively.

### The equilibrium of tetrazolo[1,5-*a*]pyrimidines in solution

**Figure 3:**
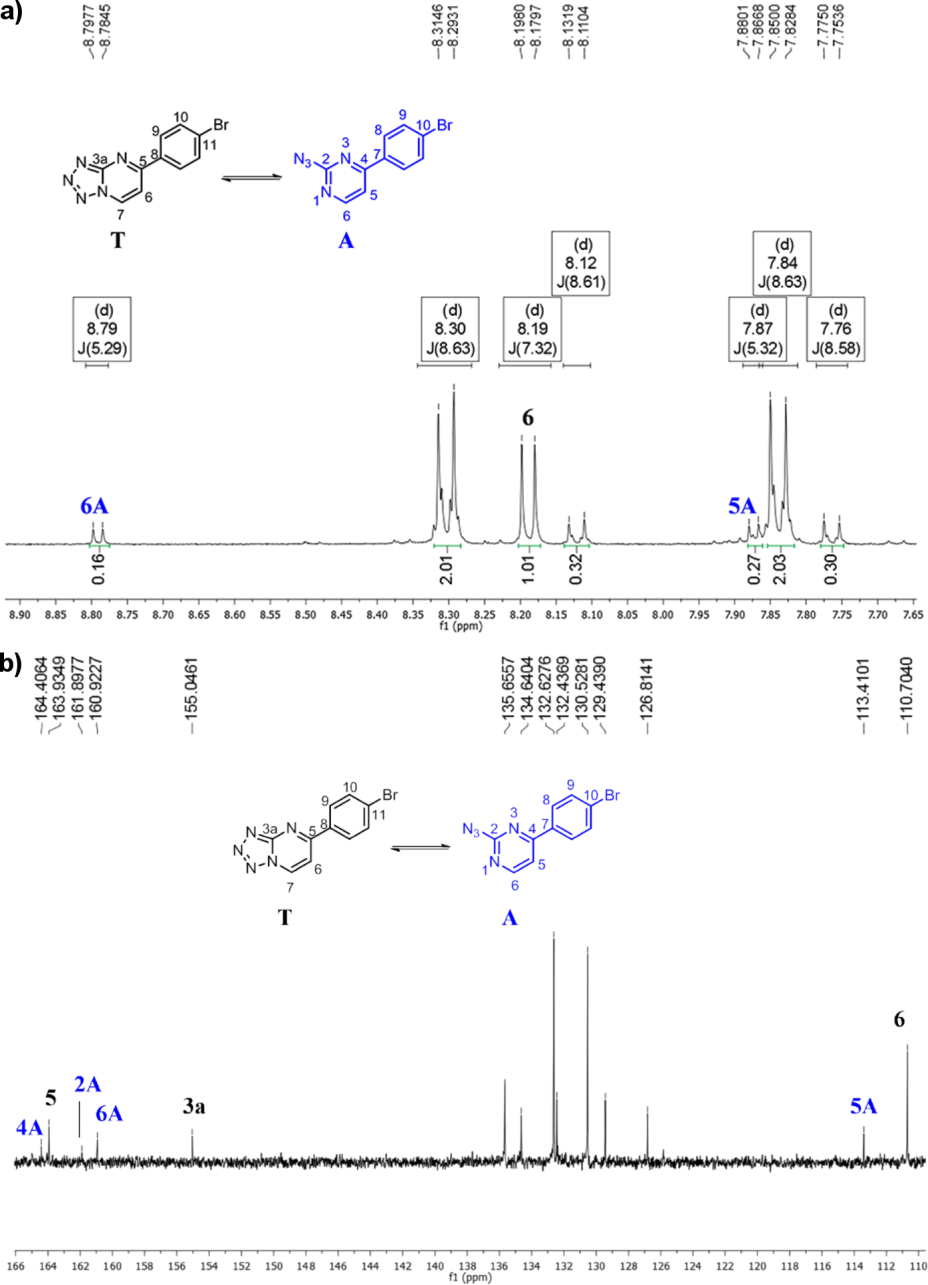
(a) ^1^H and (b) ^13^C NMR spectra demonstrating the **3d**
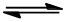
**4d** equilibrium in DMSO-*d*_6_ at 25 °C.

As previously mentioned, the products obtained from the reaction of 5-aminotetrazole with **1a**–**g** are observed as a mixture of compounds **3a**–**g**:**4a**–**g** based on the ^1^H and ^13^C NMR spectra performed in DMSO-*d*_6_. These ^1^H and ^13^C NMR analyses of **3d**:**4d** in DMSO-*d*_6_ showed a mixture of tetrazolo[1,5-*a*]pyrimidine **3d** and 2-azidopyrimidine **4d**, which suggested the existence of a tetrazolo[1,5-*a*]pyrimidine
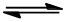
2-azidopyrimidine equilibrium (**3d**
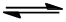
**4d**, Figure 3a,b).

In solution, the tetrazole–azido equilibrium is influenced by the polarity of the solvent. Upon increasing the solvent polarity, an increase in the amount of the tetrazole form in equilibrium is usually observed [28,36]. In general, the tetrazole form **3a**–**g** is predominant in the mixture at a proportion greater than 4:1 relative to the azide form **4a**–**g**. The molar ratio of **3a**–**g**:**4a**–**g**, which was obtained from the ^1^H NMR using DMSO-*d*_6_ as a solvent, is presented in Table 2. Although the products **3a**–**g**:**4a**–**g** have low solubility in CDCl_3_, it was possible to record the ^1^H NMR spectrum of the mixture **3d**:**4d** in this solvent. From the ^1^H NMR, it was possible to determine that over 90% of the mixture is in the form of 2-azidopyrimidine **4d** in CDCl_3_. This proves the effect of the solvent in the tetrazolo[1,5-*a*]pyrimidine
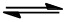
2-azidopyrimidine equilibrium. The highly polar DMSO-*d*_6_ favors the ring closure to form tetrazolo[1,5-a]pyrimidines in larger quantities (**3d**:**4d** in a proportion of 80:20). On the other hand, the less polar solvent CDCl_3_ induces the formation of 2-azidopyrimidine **4d** at a proportion of 10:90. Here, the ring is opened to form the azide group bonded in the pyrimidine ring. This result is in agreement with the literature data [20–22,24–27,32].

To corroborate with the data, the dipole moment (µ) of two forms (**3a** and **4a)** were estimated using the Gaussian 09 software package [35]. The dipole moment found for **3a** and **4a** was 7.47 D and 3.83 D, respectively. These results reveal why the tetrazole form is favored in DMSO-*d*_6_ when compared to CDCl_3_, since the dipole moment of the DMSO-*d*_6_ is greater than the dipole moment of CDCl_3,_ in addition to being able to stabilize the tetrazole form more effectively. Moreover, the results from quantum mechanical calculation confirmed that the tetrazole **3a** form is 2.98 kcal mol^−1^ more stable in DMSO-*d*_6_ when compared to its stability in CDCl_3_. A study of the azide–tetrazole equilibrium in several furo[2,3-*e*][1,5-*c*]pyrimidines was also performed by Sirakanyan et al. [36]. They showed that upon increasing the solvent polarity of CCl_4_ (ε 2.3) to DMSO-*d*_6_ (ε 46.7), the amount of the tetrazolo form increases (from 6% to 93%) in the equilibrium. These data corroborate the results herein regarding the influence of the solvent. It is important to note that the equilibrium is only established when the tetrazolo[1,5-*a*]pyrimidine is 5-substituted. 7-substituted compounds **5h**,**i** were obtained as single products.

Recently, our research group demonstrated a highly regioselective synthesis of a series of trifluoromethylated tetrazolo[1,5-*a*]pyrimidines with potent antimicrobial activity [37]. The reaction between several β-alkoxyvinyl trifluoromethyl ketones [CF_3_C(O)CH=C(R)OCH_3_] (in which R = Ph, 4-F–C_6_H_4_, 4-Cl–C_6_H_4_, 4-Br–C_6_H_4_, 4-I–C_6_H_4_, 4-CH_3_–C_6_H_4_, 4-OCH_3_–C_6_H_4_, thien-2-yl, 4-Ph–C_6_H_4_, CH_3_) and 5-aminotetrazole, by using conventional heating in an oil bath or microwave irradiation, was performed in IL. Here, only the 5-trifluoromethyl-substituted tetrazolo[1,5-*a*]pyrimidines **6a**–**g** were obtained in all cases. Some important structures for the current study are depicted in Table 3. The results were correlated with LUMO coefficient data of β-alkoxyvinyl trifluoromethyl ketones, in which the first nucleophilic attack of the 5-aminotetrazole amino group occurs in the C4 [37]. The absence of the tetrazolo[1,5-*a*]pyrimidine
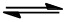
2-azidopyrimidine equilibrium in solution in CDCl_3_ or DMSO-*d*_6_, in this case, can be attributed to the presence of the CF_3_ group at the 7-position of the ring. The energy data from DFT calculations for **6a** in CDCl_3_ (8.92 kcal mol^−1^) and DMSO-*d*_6_ (7.65 kcal mol^−1^) indicate that the tetrazole form is more stable than the azide in both solvents evaluated. Unexpectedly, the tetrazole form is slightly more stable in CDCl_3_ when CX_3_ is bonded at the 7-position of the ring.

**Table 3:** Structures of trihalomethylated tetrazolo[1,5-*a*]pyrimidines.

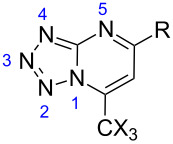		

compound	R	X

**6a**	Ph	F
**6b**	4-Br–C_6_H_4_	F
**6c**	4-OCH_3_–C_6_H_4_	F
**6d**	4-F–C_6_H_4_	F
**6e**	4-Cl–C_6_H_4_	F
**6f**	4-CH_3_–C_6_H_4_	F
**6g**	CH_3_	F
**6h**^a^	Ph	Cl
**6i**^a^	CH_3_	Cl

^a^For synthesis, see Experimental section.

Moreover, the ^15^N NMR analysis was carried out for compounds **6a** and **6g**. The spectra of the phenyl-substituted compound **6a**, in CDCl_3_, showed a signal at δ 114.8, which is consistent with sp^3^ nitrogen. This signal was attributed to N1. The N5, N4, N2, and N3 atoms appear at δ 237.1, 241.3, 252.4, and 265.7, respectively. The data obtained from the ^15^N NMR spectrum confirms the structure of trifluoromethylated tetrazolo[1,5-*a*]pyrimidines in solution (See Figures S8 and S9 in Supporting Information File 1) [38–40].

Conversely, in an attempt to extend the studies in this area and to confirm the possibility of the tetrazolo[1,5-*a*]pyrimidines
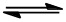
2-azidopyrimidine equilibrium for trifluoromethylated tetrazolo[1,5-a]pyrimidines in solution, three reactions were planned between phenylacetylene and compounds **6a**–**c**.

The 1,2,3-triazole synthesis from the 1,3-dipolar cycloaddition reaction between **6a**–**c** and terminal alkynes catalyzed by copper salts (CuAAC) [41–43] confirms that the reaction passes through an azide intermediate. In addition to mild reaction conditions and short reaction times, the advantage of CuAAC is the formation of 1,2,3-triazoles-1,4-disubstituted in a highly regioselective manner [41]. Recently, Cornec et al. [44] synthesized 4,6-dimethyl-2-(4-aryl-1*H*-1,2,3-triazol-1-yl)pyrimidines from azides using copper salts. The reaction involved the in situ reduction of the Cu(II) salt by sodium ascorbate.

The reactions in this study were performed from the 1,3-dipoloar cycloaddition reaction catalyzed by Cu(I) [44] and the 1,4-disubstituted 1,2,3-triazoles **8a–c** were acquired in excellent yields (Table 4). Although only compounds **6a–c** were observed in solution, the different conditions at which the compounds were introduced can induce the establishment of an equilibrium and the presence of an azide in solution. This data indicates that the equilibrium between these two are complex and depend on conditions beyond the nature of the substituent of the tetrazolo[1,5-*a*]pyrimidines and/or the solvent used to dissolve the compounds.

**Table 4:** Reaction conditions^a^ and yields^b^ of **8a–c**.

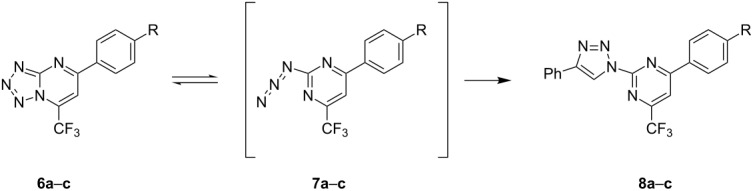		

product	R	yield (%)^b^

**8a**	H	91
**8b**	Br	94
**8c**	OCH_3_	93

^a^Reaction conditions: phenylacetylene, CuSO_4_·2H_2_O (10 mol %), sodium ascorbate (20%), *tert*-BuOH/H_2_O (1:1), 60 °C, 24 h. ^b^Isolated product yield.

### The equilibrium of tetrazolo[1,5-*a*]pyrimidine and 2-azidopyrimidine in the solid state

Furthermore, as observed in the literature, in the solid state or in DMSO-*d*_6_, the tetrazolo[1,5-*a*]pyrimidines are favored or predominant in the tetrazolo[1,5-a]pyrimidine
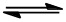
2-azidopyrimidine equilibrium [1,21–24,26–27]. Additionally, from quantum mechanical calculations, it was possible to note that compound **3a** (tetrazole form) is 1.54 kcal mol^−1^ more stable than **4a** (azide form) in the solid state.

**Figure 4:**
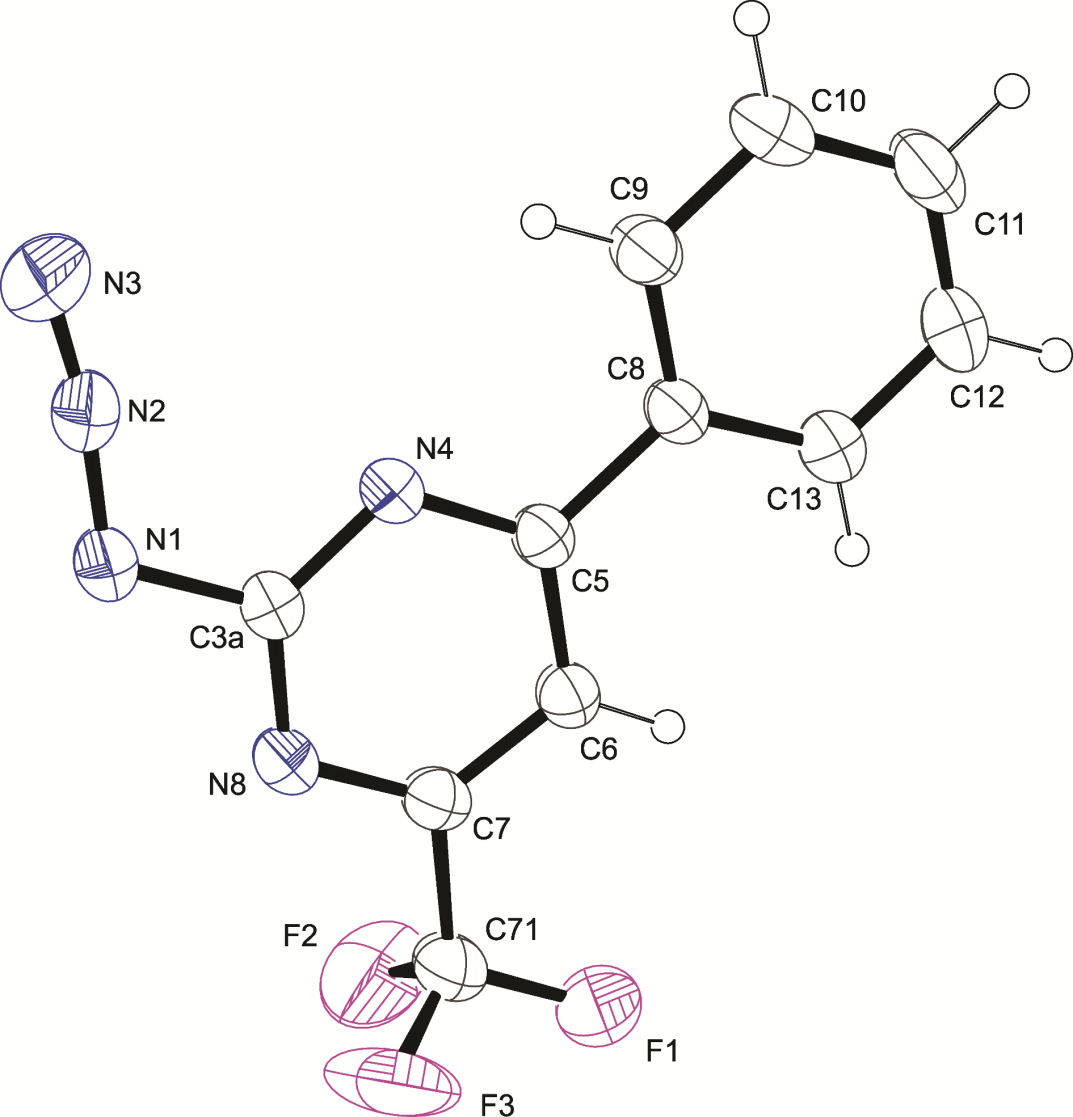
ORTEP^®^ [45] plot of **7a** with thermal ellipsoids drawn at 50% probability level.

To corroborate this result, compounds **6a**,**b**,**d**–**f** (previously described in reference [37]) were evaluated from the solid state equilibrium point of view (Figure 4). To evaluate the predominant form in the solid state, compounds **6a**,**b,d**–**f** were crystallized and single crystal X-ray diffraction (SCXRD) data were collected. In the solid state, the analysis revealed that the products **6a**,**b,d**–**f** are, in fact, in the 2-azidopyrimidine form **7a**,**b,d**–**f**. It is opposite the trend regarding compound **3a** from DFT data. The ORTEP^®^ plot of **7a** is depicted in Figure 4. The ORTEP^®^ plot of **7b** and **7d**–**f** can be observed in Figure S10 in Supporting Information File 1.

Similar to our study, Sirakanyan et al. [46] presented the synthesis and structure of condensed triazolo- and tetrazolopyrimidines, wherein, in DMSO-*d*_6_, tetrazolo-azidopyrimidines were in equilibrium. However, the structure confirmed by X-ray crystallography was the tetrazole form, which is different from our results. This demonstrates how different factors, such as the nature of the substituents and influence of solvents, can change the tetrazole–azide equilibrium.

For this series, when the compounds crystallize (solid form), the equilibrium is shifted to the opened aromatic ring, resulting in a 2-azidopyrimidine compound. The structures of compounds **7a**,**b** and **7f** were also confirmed by IR spectroscopy in the solid state. The FTIR spectrum, recorded using a KBr pellet, exhibited a strong absorption band corresponding to the azido group [47] at 2140 cm^−1^ and 2160 cm^−1^, thus corroborating the SCXRD results (see Figures S11–S13 in Supporting Information File 1).

**Figure 5:**
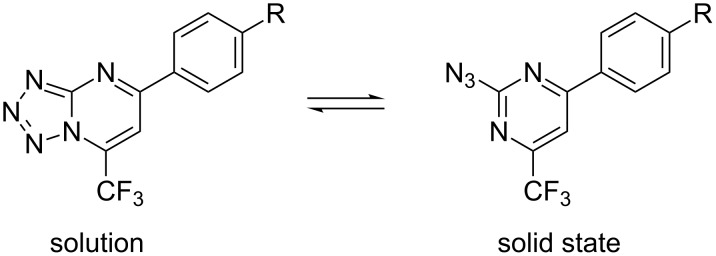
Tetrazolo[1,5-*a*]pyrimidine observed in solution (CDCl_3_ and DMSO-*d*_6_) and 2-azidopyrimidine observed in the solid state based on equilibrium.

Despite the considerable amount of experimental data reported in the literature regarding the azido–tetrazole equilibrium, quantum mechanical studies in this context have yet to be thoroughly explored. This approach provides energy data about the stabilization of the different forms, solvent effects, and shows that the azide–tetrazole transformation is initiated by a p–π atomic orbital overlap rather than an electrostatic attraction process [48]. To detect which form is more stable in the solid state, quantum mechanical calculations were performed using the Gaussian 09 software package [35] and B3LYP/cc-pVTZ level of theory for **6a** and **6g**. The results suggest that the 2-azidopyrimidine derivatives **7a**–**g** are about 10 kcal mol^−1^ more stable than the tetrazolo[1,5-*a*]pyrimidine derivatives **6a**–**g**. Figure 5 shows the trifluoromethylated tetrazolo[1,5-*a*]pyrimidine structures favored in different conditions in equilibrium.

**Figure 6:**
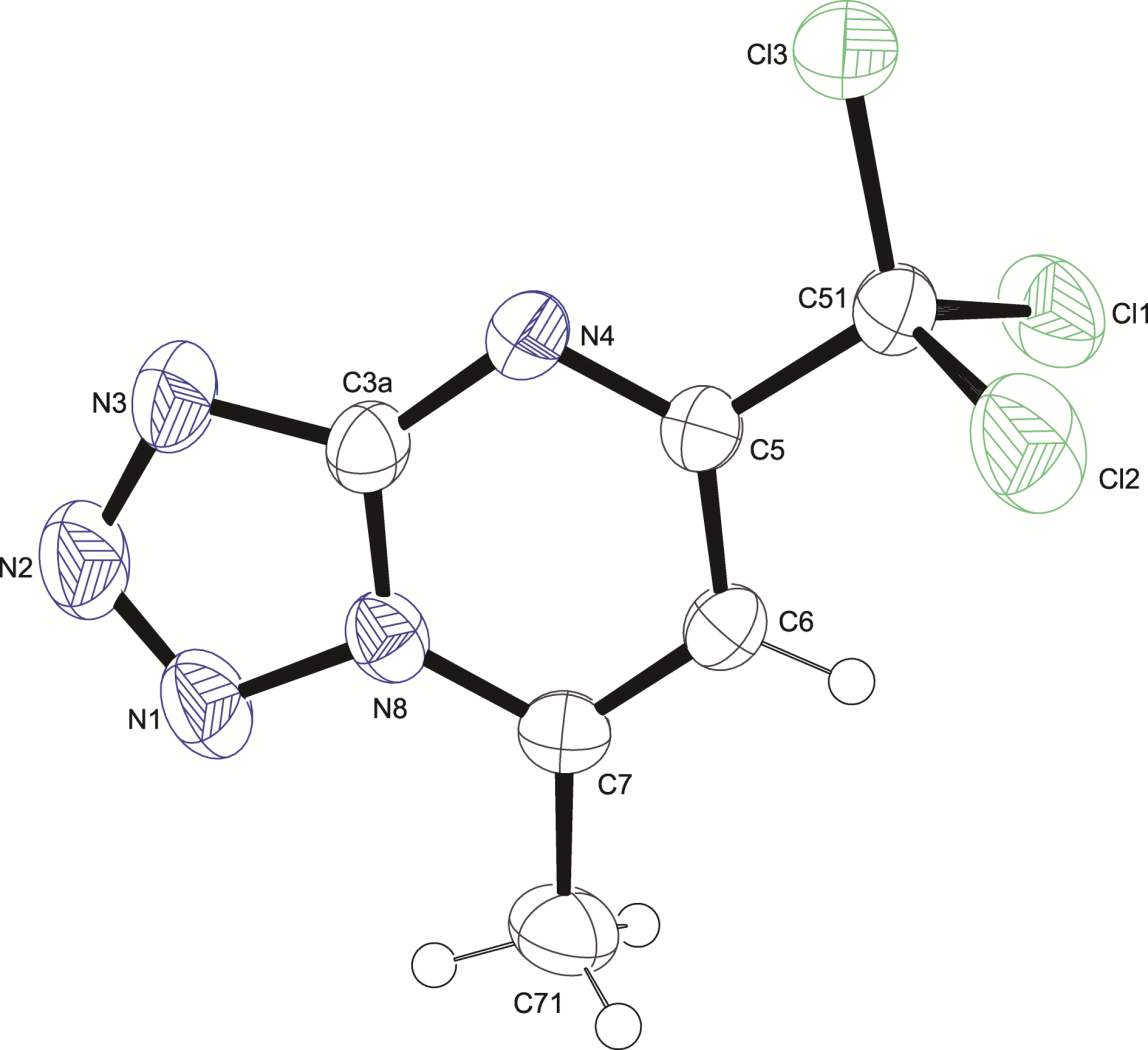
ORTEP^®^ [45] plot of **8i** with thermal ellipsoids drawn at 50% probability level.

**Figure 7:**
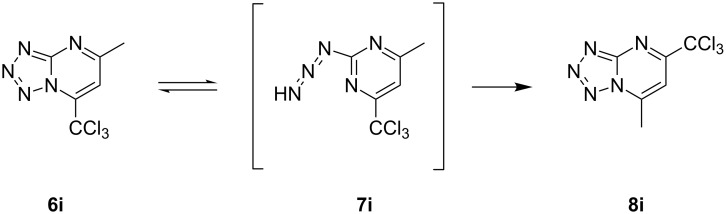
Representation of the possible equilibrium existing between **6i**, **7i**, and **8i**.

To support this data, compound **6h**,**i** (X = Cl) was synthesized using the methodology described in [37]. In a CDCl_3_ or DMSO-*d*_6_ solution, the compounds **6h**,**i** were identified as tetrazolo[1,5-*a*]pyrimidines (1,5-isomer). Surprisingly, in the solid state (after the crystallization process), compounds **6i** (or **7i** – azide form) were not observed. In this case, the 7-substituted 5-(trichloromethyl)tetrazolo[1,5-*a*]pyrimidine was found, in which the CCl_3_ group is bonded at the 5-position of the heterocyclic ring, while CH_3_ is bound at the 7-position (**8i**, Figure 6). The observation was supported by DFT data, in which the tetrazole form was found to be 2.84 kcal mol^−1^ more stable than its azide form. This indicates that an unusual equilibrium is established for **6i** (i.e., when R = CH_3_) in the crystallization process (Figure 7). Notwithstanding, **6h** was found in the usual azide form in the solid state **7h** (see Figure S10 in Supporting Information File 1).

Compound **8i** can be formed when **6i** passes through the azide intermediary **7i** (open ring) due to the presence of the **6i**:**7i** equilibrium. When the azide form **7i** reacted to tetrazolo **8i**, the nitrogen atom next to the methyl group participates in the ring closure. This equilibrium was not observed in the other compounds addressed in this work.

In general, tetrazolo[1,5-*a*]pyrimidines with 7-CF_3_ or 7-CCl_3_ substituents were observed in DMSO-*d*_6_ or CDCl_3_, while 2-azidopyrimidine was found in the solid state. As previously mentioned, the existence of the tetrazolo form in solution can be explained by the presence of electron-withdrawing groups (CX_3_) which stabilize this form. On the other hand, in solid state the azide form (more thermodynamically stable) predominates, except for **8i**.

Abu-Eittah et al. [49] theoretically investigated the substituent effect of azidothiazoles in the azido–tetrazole equilibrium using the B3LYP/6-311G** level of theory. Their results showed that electron-withdrawing groups (–NO_2_, –CN) stabilize azide isomers while –NH_2_ and –OH groups shift the equilibrium to the tetrazole side. Furthermore, energy calculations demonstrated that tetrazole is less stable than the azide isomer by an amount of energy between 16.02 and 5.44 kcal mol^−1^. In this case, compounds with NO_2_ and NH_2_ substituent has the highest and lowest value, respectively. Coutinho et al. [20] found the same tendency while studying a series of substituents at the 5-position of tetrazolo[1,5-*a*]pyridines using ab initio calculations. Their findings indicated that electron-withdrawing groups stabilize the azide isomer while electron-donating groups stabilize the tetrazole ring. Additionally, it was observed that NO_2_ has the largest effect on the equilibrium, stabilizing the azide by 7.0 kcal mol^−1^ in comparison with the tetrazole. Based on these results, our data are considered to be in accordance with the literature. Therefore, the authors suggest that the CX_3_ electron-withdrawing groups at the 7-position also present important effects to obtain azide isomers in the solid state (**7a*,*b*,*d–f**) since, with the exception of R = CH_3_ at the 5-position (**8i**), all the substituents were supplied in the azide form in the solid state.

## Conclusion

A highly regioselective synthesis of triazolo[1,5-*a*]pyrimidines according to the substituent of the β-enaminone **1** was reported. All products were obtained in good to excellent yields (40–96%) and 13 compounds were described for the first time. It was shown that aryl (electron-donating) and CX_3_ (electron-withdrawing) groups in precursor **1** lead to 5-substituted and 7-substituted triazolo[1,5-*a*]pyrimidine compounds, respectively. The results indicate that the reaction regiochemistry is governed by the group bound to the carbonyl of the β-enaminones. In addition, the tetrazole–azide equilibrium was only detected in solution (DMSO-*d*_6_) for the 1,5-isomers **3a**–**g**:**4a**–**g**. In this case, the predominant form was tetrazole. When **3d**:**4d** was evaluated in CDCl_3_, a proportion of 10:90 was observed, which corroborates with predominant forms already described in the literature for triazolo[1,5-*a*]pyrimidines in both solvents. From the DFT calculations, it was possible to predict that the tetrazole form can be expected in the solid state.

Based on these data, some important insights about the equilibrium in the solid state for 7-trihalomethyl-substituted tetrazolo[1,5-*a*]pyrimidines were also reported. Unexpectedly, these products were observed predominantly in the tetrazole form **6a**–**h** in solution (CDCl_3_ and DMSO-*d*_6_), while azide species **7a**–**h** were observed in the solid state. Notwithstanding, although only the tetrazoles were observed for compounds **6a**–**c** in solution, the conditions at which the compounds were introduced can induce the equilibrium to favor the presence of the azides (e.g., reaction conditions to achieve **8a**–**c**). In the solid state, the DFT results revealed that the 2-azidopyrimidine derivatives **7a**–**h** are approximately 10 kcal mol^−1^ more stable than tetrazolo[1,5-*a*]pyrimidine derivatives **6a**–**h**, which is consistent with the data obtained. This behavior was related to the presence of the CX_3_ group at the 7-position of the ring. In addition, an unusual equilibrium was observed for compound **6i**. The result of **8i** from **6i** crystallization was attributed to the equilibrium **6i**
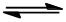
**7i** (in which **7i** is the azide form).

Lastly, the evaluated series were very important to elucidate the factors that affect the equilibrium in distinct types of tetrazolo[1,5-*a*]pyrimidines in solution or solid state. These results support the substituent influence in both regioselectivity and tetrazole–azide equilibrium of tetrazolo[1,5-*a*]pyrimidines with great interest to biological or material sciences.

## Experimental

The reagents and solvents used were obtained from commercial suppliers without further purification. ^1^H and ^13^C NMR spectra were recorded on Bruker DPX 400 (^1^H at 400.13 MHz and ^13^C at 100.62 MHz) and Bruker DPX-200 (^1^H at 200.13 MHz and ^13^C at 50.32 MHz) spectrometers in CDCl_3_/TMS solutions at 298 K and in DMSO-*d*_6_/TMS solutions at 298 K. All spectra were acquired in a 5 mm tube at natural abundance. The chemical shifts (δ) are reported in ppm and *J* values are given in Hz. The melting points were measured using a Microquímica MQAPF 301 apparatus. The ionic liquid 1-butyl-3-methylimidazolium tetrafluoroborate [BMIM][BF_4_] was obtained commercially. The ionic liquid [HMIM][TsO] was prepared according to procedures described in the literature [50]. Additional information regarding the experimental data for the synthesized compounds is presented in Supporting Information File 1.

### General procedure for obtaining single crystals

The single crystals of the compounds were obtained by slow evaporation of the solvents at 25 °C. Compounds **6a**,**b**,**f**,**h**, and **8i** were obtained from the solvent CHCl_3_. Suitable monocrystals for compounds **6d**,**e** were obtained from solvent mixtures of ethyl acetate and EtOH (3:2).

### General procedure for the synthesis of β-enaminones **1a**–**i**

β-Enaminones **1a**–**i** were prepared from the reaction of *N,N*-dimethylformamide dimethylacetal with methyl ketones in accordance with the methodology developed in our laboratory [31].

### General procedure for the synthesis of tetrazolo[1,5-*a*]pyrimidines **3a**–**g**, **4a**–**g**, and **5h**,**i**

A mixture of the 5-aminotetrazole (1.0 mmol) and the precursor β-enaminones **1** (1.0 mmol) in [HMIM][TsO] (1.0 mmol) and HCl (0.1 mmol) were placed in a round-bottomed flask and magnetically stirred for 5 min at 120 °C. After cooling, the resulting solid product was washed with water and collected by filtration using a funnel. The obtained products **3a**–**g**, **4a**–g**,** and **5h**,**i** were dried using a vacuum pump. Synthesis procedures and experimental data for **3a**, **3g**, **4a**, **4g,** and **5h** can also be found in [21,32].

### General procedure for the synthesis of (trichloromethyl)tetrazolo[1,5-*a*]pyrimidines **6h**,**i**

In a manner closely related to a procedure from [37], a mixture of the 5-aminotetrazole (1.0 mmol) and the precursor β-enaminones **1** (1.0 mmol) in [BMIM][BF_4_] (1.0 mmol) and HCl (0.1 mmol) were placed in a round-bottomed flask and magnetically stirred for 6 h at 120 °C. After the reaction time, chloroform (30 mL) was added and the resulting mixture was washed with distilled water (3 × 10 mL), dried over sodium sulfate (Na_2_SO_4_), and the solvent was then removed under reduced pressure. The compounds **6h**,**i** were obtained in pure form.

### General procedure for the synthesis of tetrazolo[1,5-*a*]pyrimidines **8a**–**c**

A mixture of 5-aryl-7-trifluoromethyltetrazolo[1,5-*a*]pyrimidine **6a**–**c** (1.0 mmol), phenylacetylene (1 mmol), copper sulfate pentahydrate (10 mol %), and sodium ascorbate (20 mol %) in *tert*-butyl alcohol/water (1:1 mL) were placed in a round-bottomed flask and magnetically stirred at 60 °C for 24 h. After the reaction time was reached, chloroform (30 mL) was added and the resulting mixture was washed with distilled water (3 × 10 mL), dried over sodium sulfate (Na_2_SO_4_), and the solvent was then removed under reduced pressure. The compounds **8a–c** were obtained in pure form.

## Supporting Information

File 1 Additional material.

## References

[1] Raju C, Madhaiyan K, Uma R, Sridhar R, Ramakrishna S (2012). RSC Adv.

[2] Aly A A (2006). Phosphorus, Sulfur Silicon Relat Elem.

[3] Nagai S-I, Ueda T, Sugiura S, Nagatsu A, Murakami N, Sakakibara J, Fujita M, Hotta Y (1998). J Heterocycl Chem.

[4] Uehata M, Ono T, Satoh H, Yamagami K, Kawahara T (2001). Medicines Comprising Rho Kinase Inhibitor. U.S. Patent.

[5] Aspnes G E, Chiang Y-C P (2002). Tetrazole Compounds as Thyroid Receptor Ligands. U.S. Patent.

[6] Fujii A, Tanaka H, Otsuki M, Kawaguchi T, Oshita K (2005). Antitumor effect potentiators. U.S. Patent.

[7] Takayama Y, Yoshida Y, Uehata M (2006). Visual Function Disorder Improving Agents. U.S. Patent.

[8] Hussein A M, Ahmed O M (2010). Bioorg Med Chem.

[9] Hussein A M (2010). J Saudi Chem Soc.

[10] Takao Takaya K, Masayoshi Murata O, Kiyotaka Ito I (1988). Pyrimidine Compounds Having Activity as a Cardiotonic Anti-Hypertensive Cerebrovascular Vasodilator and Anti-Platelet Aggregation Agent. U.S. Patent.

[11] Utsunomiya T, Niki T, Kikuchi T, Watanabe J, Yamagishi K, Nishioka M, Suzuki H, Furusato T, Miyake T (1999). Tetrazole Compounds and Pest Control Agent. WO Patent.

[12] Abelman M, Jiang R, Zablocki J (2009). Substituted heterocyclic compounds. U.S. Patent.

[13] Dougherty A M, Guo H, Westby G, Liu Y, Simsek E, Guo J-T, Mehta A, Norton P, Gu B, Block T (2007). Antimicrob Agents Chemother.

[14] Raju C, Kalaipriya M, Uma R, Sridhar R, Ramakrishna S (2012). Curr Chem Lett.

[15] Zeng L-Y, Cai C (2010). J Comb Chem.

[16] Yao C, Lei S, Wang C, Yu C, Tu S (2008). J Heterocycl Chem.

[17] Ghorbani-Vaghei R, Toghraei-Semiromi Z, Amiri M, Karimi-Nami R (2013). Mol Diversity.

[18] Desenko S M, Gladkov E S, Komykhov S A, Shishkin O V, Orlov V D (2001). Chem Heterocycl Compd.

[19] Gein V L, Gein L F, Tsyplyakova E P, Panova O S (2007). Russ J Org Chem.

[20] Kanyalkar M, Coutinho E C (2000). Tetrahedron.

[21] Krivopalov V P, Mamatyuk V I, Nikolaenkova E B (1995). Russ Chem Bull.

[22] Bajwa J S, Sykes P J (1979). J Chem Soc, Perkin Trans 1.

[23] Cubero E, Orozco M, Luque F J (1998). J Am Chem Soc.

[24] Temple C, McKee R L, Montgomery J A (1962). J Org Chem.

[25] Boyer J, Hyde H (1960). J Org Chem.

[26] Temple C, Thorpe M C, Coburn W C, Montgomery J A (1966). J Org Chem.

[27] Temple C, Montgomery J A (1965). J Org Chem.

[28] Pochinok V Y, Avramenko L F, Grigorenko P S, Skopenko V N (1975). Russ Chem Rev.

[29] Martins M A P, Scapin E, Frizzo C P, Rosa F A, Bonacorso H G, Zanatta N (2009). J Braz Chem Soc.

[30] Frizzo C P, Scapin E, Marzari M R B, München T S, Zanatta N, Bonacorso H G, Buriol L, Martins M A P (2014). Ultrason Sonochem.

[31] Martins M A P, Frizzo C P, Moreira D N, Rosa F A, Marzari M R B, Zanatta N, Bonacorso H G (2008). Catal Commun.

[32] Krasovsky A L, Moiseev A M, Nenajdenko V G, Balenkova E S (2002). Synthesis.

[33] Shao Y, Zhu K, Qin Z, Li E, Li Y (2013). J Org Chem.

[34] Xu H, Zhou B, Zhou P, Zhou J, Shen Y, Yu F-C, Lu L-L (2016). Chem Commun.

[35] (2016). Gaussian 09.

[36] Sirakanyan S N, Spinelli D, Geronikaki A, Kartsev V G, Panosyan H A, Ayvazyan A G, Tamazyan R A, Frenna V, Hovakimyan A A (2016). Tetrahedron.

[37] Scapin E, Frizzo C P, Rodrigues L V, Zimmer G C, Vaucher R A, Sagrillo M R, Giongo J L, Afonso C A M, Rijo P, Zanatta N (2017). Med Chem Res.

[38] Shestakova T S, Shenkarev Z O, Deev S L, Chupakhin O N, Khalymbadzha I A, Rusinov V L, Arseniev A S (2013). J Org Chem.

[39] Cmoch P, Stefaniak L, Webb G A (1997). Magn Reson Chem.

[40] Cmoch P (2002). Magn Reson Chem.

[41] Rostovtsev V V, Green L G, Fokin V V, Sharpless K B (2002). Angew Chem, Int Ed.

[42] Hein J E, Fokin V V (2010). Chem Soc Rev.

[43] Tornøe C W, Christensen C, Meldal M (2002). J Org Chem.

[44] Cornec A-S, Baudequin C, Fiol-Petit C, Plé N, Dupas G, Ramondenc Y (2013). Eur J Org Chem.

[45] Farrugia L J (1997). J Appl Crystallogr.

[46] Sirakanyan S N, Geronikaki A, Spinelli D, Hovakimyan A A, Noravyan A S (2013). Tetrahedron.

[47] Bajwa J S, Sykes P J (1980). J Chem Soc, Perkin Trans 1.

[48] Abu-Eittah R H, El-Taher S, Hassan W M I, Noamaan M A (2014). Comput Theor Chem.

[49] Abu-Eittah R H, El-Kelany K E (2012). Spectrochim Acta, Part A.

[50] Zhao G, Jiang T, Gao H, Han B, Huang J, Sun D (2004). Green Chem.

