# Supplementary material for: Synthesis, effect of substituents on the regiochemistry and equilibrium studies of tetrazolo[1,5-a]pyrimidine/2-azidopyrimidines
Source: Beilstein J Org Chem. 2017 Nov 10;13:2396–407. doi: 10.3762/bjoc.13.237 (PMC5687014; doi:10.3762/bjoc.13.237)
Supplement: File 1 — Additional material. [file Beilstein_J_Org_Chem-13-2396-s001.pdf]

Supporting Information

for

Synthesis, effect of substituents on the regiochemistry and equilibrium studies of tetrazolo[1,5-*a*]pyrimidine/2-azidopyrimidines

Elisandra Scapin[1], Paulo R. S. Salbego[2], Caroline R. Bender[2], Alexandre R. Meyer[2], Anderson B. Pagliari[2], Tainára Orlando[2], Geórgia C. Zimmer[2], Clarissa P. Frizzo[2], Helio G. Bonacorso[2], Nilo Zanatta[2] and Marcos A. P. Martins[2]*

Address: [1]Laboratório de Química, Universidade Federal do Tocantins, Palmas, TO 77001-090, Brazil and [2]Núcleo de Química de Heterociclos (NUQUIMHE), Department of Chemistry, Federal University of Santa Maria (UFSM), 97105-900, Santa Maria, RS, Brazil

Email: Marcos A. P. Martins - marcos.nuquimhe@gmail.com

*Corresponding author

Additional material

1. Experimental data for the synthesized compounds S2
2. Tetrazolo[1,5-*a*]pyrimidines synthesis S4
3. Regiochemistry of the reactions according with β-enaminones substituent S4
4. The equilibrium of tetrazolo[1,5-*a*]pyrimidines in solution S7
5. ORTEP and crystallographic data S9
6. HOMO coefficients S12
7. FTIR spectrum of compounds **7a,b** and **7f** S12
8. Mechanism of trifluoromethylated tetrazolo[1,5-*a*]pyrimidines (**6a–i**) formation. S13
9. NMR spectrum of compound **3b–f**, **5i**, **6h–i** and **8a–c** S14

1. Experimental data for the synthesized compounds

5-Phenyltetrazolo[1,5-a]pyrimidine (**3a**) and *2-azido-4-phenylpyrimidine* (**4a**): $C_{10}H_7N_5$ (Mw. 197.07). Yield 82%; m.p. 129-131°C. ^{1}H NMR (400 MHz, DMSO-d_6) for compound **3a**: δ= 9.82 (d, ^3J 7, 1H, H7),8.40-8.36 (m, 2H, H-Ar), 8.24 (d, ^3J 7, 1H, H6), 7.73-7.59 (m, 3H, H-Ar). ^{13}C NMR (100 MHz, DMSO-d_6): δ = 165.0 (C5),155.2 (C3a), 135.6 (C7), 132.8, 129.7, 128.7, 127.5 (C-Ar), 110.9 (C6). ^{1}HNMR (400 MHz, DMSO-d_6) for compound **4a**: δ= 8.79 (d, ^3J 5, 1H, H6), 7.89 (d, ^3J 5, 1H, H5).^{13}C NMR (100 MHz, DMSO-d_6): δ= 165.5 (C4), 160.8 (C2), 135.5 (C6), 132.1, 130.0, 129.5, 127.5 (C-Ar), 113.5 (C5). LCMS MS: = 198.2 $[M+H]^+$.

5-(4-Fluorophenyl)tetrazolo[1,5-a]pyrimidine (**3b**) and *2-azido-4-(4-fluorophenyl)pyrimidine* (**4b**): $C_{10}H_6FN_5$ (Mw. 215.06). Yield 83%; m.p. 122-124°C. ^{1}H NMR (400 MHz, DMSO-d_6) for compound **3b**: δ= 9.80 (d, ^3J 7, 1H, H7), 8.46-8.42 (m, 2H, H-Ar), 8.21 (d, ^3J 7, 1H, H6), 7.46 (t, 2H, H-Ar). ^{13}C NMR (100 MHz, DMSO-d_6): δ= 163.8 (C5), 155.1 (C3a), 135.7 (C7), 132.0 (^3J 3), 131.4 (^2J 9.2), 116.8 (^1J 21.9) (C-Ar),110.8 (C6). ^{1}H NMR (400 MHz, DMSO-d_6) compound **4b**: δ= 8.76 (d, ^3J 5, 1H, H6), 8.25 (d, 2H, H-Ar), 7.86 (d, ^3J 5, 1H, H5), 7.36 (d, 2H, H-Ar). ^{13}C NMR (100 MHz, DMSO-d_6): δ= 166.9 (C4), 163,6 (C2),160.9 (C6),166.4 (^3J 2), 130.1 (^2J 9) (C-Ar),113.3 (C5). LCMS MS: = 216.1 $[M+H]^+$. Anal. Cald. for $C_{10}H_6FN_5$ (M+H): 216.0685; found: 216.0664.

5-(4-Chlorophenyl)tetrazolo[1,5-a]pyrimidine (**3c**) *2-azido-4-(4-chlorophenyl)pyrimidine* (**4c**): $C_{10}H_6ClN_5$ (Mw. 231.03). Yield 91%; m. p. 138-140 °C. ^{1}H NMR (400 MHz, DMSO-d_6) for compound **3c**: δ = 9.69 (d, ^3J 7, 1H, H7), 8.30 (d, ^3J 8, 2H, H-Ar), 8.11 (d, ^3J 7, 1H, H6), 7.63 (d, ^3J 8, 2H, H-Ar). ^{13}C NMR (100 MHz, DMSO-d_6): δ = 163.9 (C5), 155.0 (C3a), 135.6 (C7), 134.2, 130.4, 129.7, 129.2 (C-Ar), 110.8 (C6). ^{1}H NMR (400 MHz, DMSO-d_6) for compound **4c**: δ = 8.72 (d, ^3J 5, 1H, H7), 7.78 (d, ^3J 5, 1H, H6), 7.72 (d, ^3J 8, 2H, H-Ar), 7.54 (d, ^3J 8, 2H, H-Ar).^{13}C NMR (100 MHz, DMSO-d_6): δ = 164.3 (C4), 163.9 (C2), 160.9 (C6), 137.9, 137.0, 129.7, 129.5 (C-Ar), 114.4 (C5). MS (EI, 70 eV): *m/z* % = 231 ((M^+,92), 203 (100), 176.7 (28), 82.6 (27). Anal. Cald. for $C_{10}H_6FN_5$ (M+H): 232.0390; found: 232.0384.

5-(4-Bromophenyl)tetrazolo[1,5-a]pyrimidine **(3d)** and*2-azido-4-(4-bromophenyl)pyrimidine* **(4d)**: $C_{10}H_6BrN_5$ (Mw. 274.98). Yield 95%; 147-149°C. ^{1}H NMR (400 MHz, DMSO-d_6) for compound **3d**: δ = 9.79 (d, ^3J 7, 1H, H7), 8.31 (d, ^3J 9, 2H, H-Ar), 8.19 (d, ^3J 7, 1H, H6), 7.84 (d, ^3J 9, 2H, H-Ar). ^{13}C NMR (100 MHz, DMSO-d_6): δ = 163.6 (C5), 154.7 (C3a), 135.2 (C7), 132.2, 130.1, 129.0, 126.5 (C-Ar), 110.8 (C6). ^{1}H NMR (400 MHz, DMSO-d_6) for compound **4d**: δ = 8.79 (d, ^3J 5, 1H, H6), 8.12 (d, ^3J 9, 2H, H-Ar), 7.87 (d, ^3J 5, 1H, H5), 7.77 (d, ^3J 8.6, 2H, H-Ar). ^{13}C NMR (100 MHz, DMSO-d_6): δ = 163.0 (C4), 161.5 (C2), 160.5 (C6), 134.2, 132.2, 130.2, 125.5 (C-Ar), 113.5 (C5). LCMS MS: = 276.1 $[M+H]^+$. Anal. Cald. for $C_{10}H_6BrN_5$ (M+H): 275.9885; found: 275.9870.

5-(4-Iodophenyl)tetrazolo[1,5-a]pyrimidine **(3e)** and*2-azido-4-(4-iodophenyl)-pyrimidine* **(4e)**:$C_{10}H_6IN_5$ (Mw. 322.97). Yield 86%; 167-199°C. ^{1}H NMR (400 MHz, DMSO-d_6) for compound **3e**: δ = 9.78 (d, ^3J 7, 1H, H7), 8.18 (d, ^3J 7, 1H, H6), 8.12 (d, ^3J 8, 2H, H-Ar), 8.00 (d, ^3J 8, 2H, H-Ar). ^{13}C NMR (100 MHz, DMSO-d_6): δ = 164.2 (C5), 155.1 (C3a), 135.7 (C7), 138.6, 135.8, 130.4, 129.4, 101.1, (C-Ar), 110.7 (C6). ^{1}H NMR (400 MHz, DMSO-d_6) for compound **4e**: δ = 8.77 (d, ^3J 5, 1H, H6), 7.90 (d, ^3J 8, 2H, H-Ar), 7.85 (d, ^3J 5, 1H, H5), 7.69 (d, ^3J 8, 2H, H-Ar).^{13}C NMR (100 MHz, DMSO-d_6): δ = 164.7 (C4), 162.2 (C2), 161.0 (C6), 138.4, 135.0, 129.4, 99.8 (C-Ar), 113.4 (C5). LCMS MS: = 324 $[M+H]^+$.

5-(4-Methylphenyl)tetrazolo[1,5-a]pyrimidine **(3f)** and*2-azido-4-(4-methylphenyl)pyrimidine* **(4f)**: $C_{11}H_9N_5$ (Mw. 211.09). Yield 89%; 119-121°C. ^{1}H NMR (400 MHz, DMSO-d_6) for compound **3f**: δ = 9.72 (d, ^3J 7, 1H, H7), 8.22 (d, *J* 8, 2H, Ar), 8.13 (d, ^3J 7, 1H, H6), 7.37 (d, ^3J 8, 2H, Ar), 2.38 (s, 3H, CH_3). ^{13}C NMR (100 MHz, DMSO-d_6): δ = 164.8 (C5), 155.2 (C3a),

143.3 (C7), 135.3, 132.7, 130.3, 128.6 (C-Ar), 110.6 (C6), 21.5 (CH_3). ^{1}H NMR (400 MHz, DMSO-d_6) for compound **4f**: 2.48 (s, 3H, CH_3). ^{13}C NMR (100 MHz, DMSO-d_6): δ = 165.4 (C4), 160.6 (C2), 142.3 (C6), 130.1, 129.7, 129.3, 127.7 (C-Ar), 113.1 (C5), 31.0 (CH_3). LCMS MS: = 212.1 $[M+H]^+$. Anal. Cald. for $C_{11}H_9N_5$: C, 62.55; H, 4.29; N, 33.16; found: C, 62.64; H, 4.24; 32.86.

5-(4-Methoxyphenyl)tetrazolo[1,5-a]pyrimidine **(3g)** and *2-azido-4-(4-methoxyphenyl)pyrimidine* **(4g)**: $C_{11}H_9N_5O$ (Mw. 227.08). Yield 93%; 161-163°C. ^{1}H NMR (400 MHz, DMSO-d_6) for compound **3g**: δ = 9.69 (d, ^3J 7, 1H, H7), 8.34 (d, J 9, 2H, Ar), 8.14 (d, ^3J 7, 1H, H6), 7.14 (d, ^3J 9, 2H, Ar), 3.86 (s, 3H, OCH_3). ^{13}C NMR (100 MHz, DMSO-d_6): δ = 163.4 (C5), 155.3 (C3a), 135.1 (C7), 130.7, 129.3, 127.8, 115.2 (C-Ar), 110.4 (C6), 56.0 (OCH_3). ^{1}H NMR (400 MHz, DMSO-d_6) for compound **4g**: δ = 8.67 (d, ^3J 5.6, 1H, H6), 8.26 (d, ^3J 8.8, 2H, Ar), 7.79 (d, ^3J 5.3, 1H, H5), 7.05 (d, ^3J 8.8, 2H, Ar), 3.82 (s, 3H, OCH_3). ^{13}C NMR (100 MHz, DMSO-d_6): δ = 167.4 (C4), 160.3 (C2), 155.3 (C6), 134.3, 115.0 (C-Ar), 112.7 (C5), 55.9 (OCH_3). LCMS MS: = 228.1 $[M+H]^+$.

7-(Trifluoromethyl)tetrazolo[1,5-a]pyrimidine **(5h)** $C_5H_2F_3N_5$ (Mw. 189.10). Yield 70% (IL) and 40% (toluene). m.p. 120-122°C. ^{1}H NMR (400 MHz, $CDCl_3$: δ = 8.85 (d, ^3J 5, 1H, H6), 7.38 (d, ^3J 5, 1H, H5).^{13}C NMR (100 MHz, $CDCl_3$): δ = 163.2 (C5), 161.5 (C3a), 157.7 (q, ^2J 36, C7), 119.8 (q, ^1J 275, CF_3), 112.3 (q, ^3J2,C6).LCMS MS: = 189 $[M+H]^+$.

7-(Trichloromethyl)tetrazolo[1,5-a]pyrimidine **(5i)**: $C_5H_2Cl_3N_5$ (Mw. 238.46). Yield 68% (IL) and 77% (toluene). Oil. ^{1}H NMR (400 MHz, $CDCl_3$): δ = 8.80 (d, ^3J 5, 1H, H6), 7.67 (d, ^3J 5, 1H, H5). ^{13}C NMR (100 MHz, $CDCl_3$): δ = 168.0 (C5), 162.3 (C3a), 161.5 (C7), 111.3 (C6), 94.8 (CCl_3).LCMS MS: 238 $[M+H]^+$.

5-Phenyl-7-(trichloromethyl)tetrazolo[1,5-a]pyrimidine **(6h)**: $C_{11}H_6Cl_3N_5$ (Mw. 314.56). Yield 82%; 125-126°C. ^{1}H NMR (400 MHz, $CDCl_3$: δ = 7.50 (s, 1H, H6), 2.61 (s, 3H, CH_3). ^{13}C NMR (100 MHz, $CDCl_3$): δ = 172.9 (C5), 167.4 (C3a), 161.9 (C7), 110.7 (C6), 94.9 (CCl_3), 24.4 (CH_3).MS (EI, 70 eV): 313 (27), 250 (100), 187 (29), 77 (37).Anal. Cald.: C, 42.00; H, 1.92; N, 22.26; found: C, 42.66; H, 1.85; N, 21.12.

5-methyl-7-(trichloromethyl)tetrazolo[1,5-a]pyrimidine **(6i)**: $C_6H_4Cl_3N_5$ (Mw. 252.49). Yield 74%;83°C. ^{1}H NMR (400 MHz, $CDCl_3$: δ = 8.14-8.19 (m,2H, H-Ar),8.06 (s, 1H, H6), 7.50-7.60(m, 3H, H-Ar).^{13}C NMR (100 MHz, $CDCl_3$): δ = 168.9 (C5), 168.6 (C3a), 162.4 (C7), 135.1, 132.3, 129.1, 127.6 (C-Ar),106.9 (C6), 95.2 (CCl_3).MS (EI, 70eV): 251.9 (58), 215.9 (100). Anal. Cald.: C, 28.54; H, 1.60; N, 27.74; found: C, 28.72; H, 1.87; N, 27.71.

4-Phenyl-2-(4-phenyl-1H-1,2,3-triazol-1-yl)-6-(trifluoromethyl)pyrimidine **(8a)**: $C_{19}H_{12}F_3N_5$ (Mw. 367.33). Yield 91%; 179-181°C. ^{1}H NMR (400 MHz, $CDCl_3$): δ = 8.88 (s, 1H, H5'), 8.30 (m, 2H, H-Ar), 8.07 (s, 1H, H5), 8.00 (d, 2H, ^3J 7, H-Ar), 7.59-7.67 (m, 3H, H-Ar), 7.49 (t, 2H, H-Ar), 7,40 (t, 1H, H-Ar),7.29 (dd, 2H, ^3J 8, ^3J 9, H-Ar).^{13}C NMR (100 MHz, $CDCl_3$): δ = 168.4 (C4), 158.3 (q, ^2J 37, C6), 154.9 (C2), 148.3 (C4'), 165.9 (d, ^1J 255, CAr), 130.4 (d, ^3J 3), 130.3 (d, ^2J 9), 129.6, 128.9, 128.8, 126.1, 120.1 (q, ^1J 275.6, CF_3), 118.5 (C5'), 111.0 (C5). LCMS MS: = 368.2 $[M+H]^+$. Anal. Cald.: C, 62.13; H, 3.29; N, 19; found: C, 61.76; H, 3.42; N, 18.63.

4-(4-Bromophenyl)-2-(4-phenyl-1H-1,2,3-triazol-1-yl)-6-(trifluoromethyl)pyrimidine **(8b)**: Yield 93%; 177-179°C. ^{1}H NMR (400 MHz, $CDCl_3$): δ = 8.84 (s, 1H, H5'), 8.06 (d, ^3J 3, 1H, H-Ar), 7.99 (d, 2H, ^3J 7, H-Ar), 7.85 (s, 1H, H5), 7.74 (d, ^3J 4, 1H, H-Ar), 7.49 (t, ^3J 7, 2H, H-Ar), 7.40 (t, ^3J 7, 1H, H-Ar), 7.28 (d, ^3J 4,1H, H-Ar). ^{13}C NMR (100 MHz, $CDCl_3$): δ = 168.5 (C4), 158.4 (q, ^2J 37, C6), 154.9 (C2), 148.4 (C4'), 133.0 (CAr), 132.7, 129.5, 129.3, 128.9, 128.8, 128.3, 126.1, 120.1 (q, ^1J 276, CF_3), 118.6 (C5'), 111,2 (q, ^3J 2,5, C5). HRMS (ESI-TOF) *m/z*: $[M + H]^+$ Calcd., for $C_{19}H_{12}BrF_3N_5$ 446.0228, found: 446.0231.

4-(4-Methoxyphenyl)-2-(4-phenyl-1H-1,2,3-triazol-1-yl)-6-(trifluoromethyl)pyrimidine **(8c)**: Yield 93%; 132-134°C. ^{1}H NMR (400 MHz, $CDCl_3$): δ = 8.81 (s, 1H, H5'), 8.21 (d, ^{3}J 9, 2H, H-Ar), 7.95 (d, ^{3}J 7, 2H, H-Ar), 7.91 (s, 1H, H5), 7.48-7.35 (m, 3H, H-Ar), 7.01 (d, ^{3}J 9, 2H, H-Ar), 3.87 (s, 3H, OCH_3).^{13}C NMR (100 MHz, $CDCl_3$): δ = 168.8 (C4), 163.9 (C2), 157.7 (q, ^{2}J 36, C6), 154.8 (C4'), 129.8, 129.7, 128.8, 128.6, 126.6, 126.1 (C-Ar), 120.3 (q, ^{1}J 275, CF_3), 114.8 (C5'), 110.3 (q, ^{3}J 2, C5), 55.5 (OCH_3). HRMS (ESI-TOF) *m/z*: $[M + H]^+$ Calcd., for $C_{20}H_{14}F_3N_5O$398.1229, found: 398.1233.

2. Tetrazolo[1,5-*a*]pyrimidines synthesis

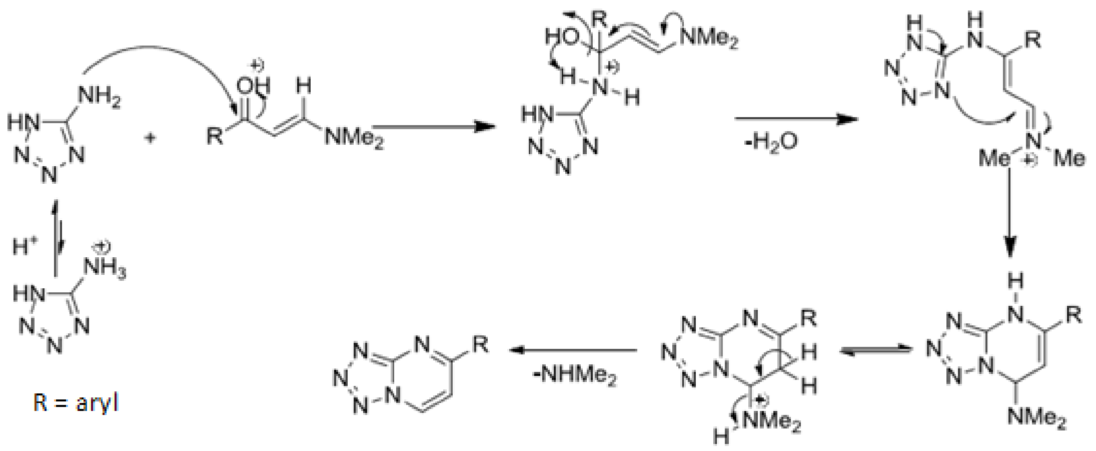

Figure S1: Scheme that illustrate the mechanism of tetrazolo[1,5-*a*]pyrimidines formation from β-enaminone and 5-aminotetrazole.

3. Regiochemistry of the reactions according with β-enaminones substituent

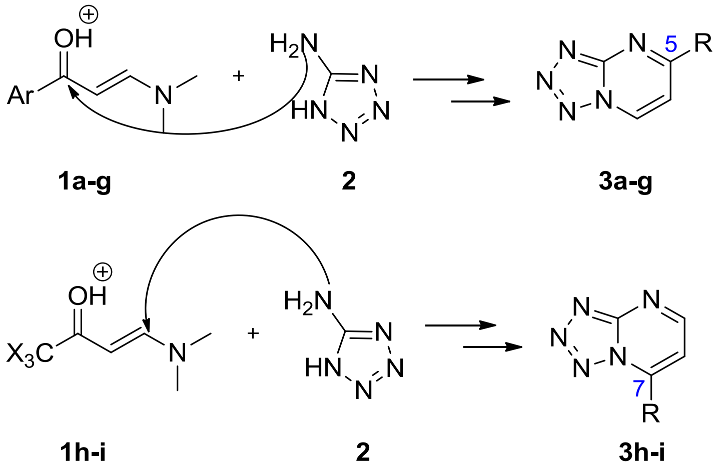

Figure S2: The scheme illustrate the first nucleophilic attack which lead to different tetrazolo[1,5-*a*]pyrimidines, according with β-enaminone substituent.

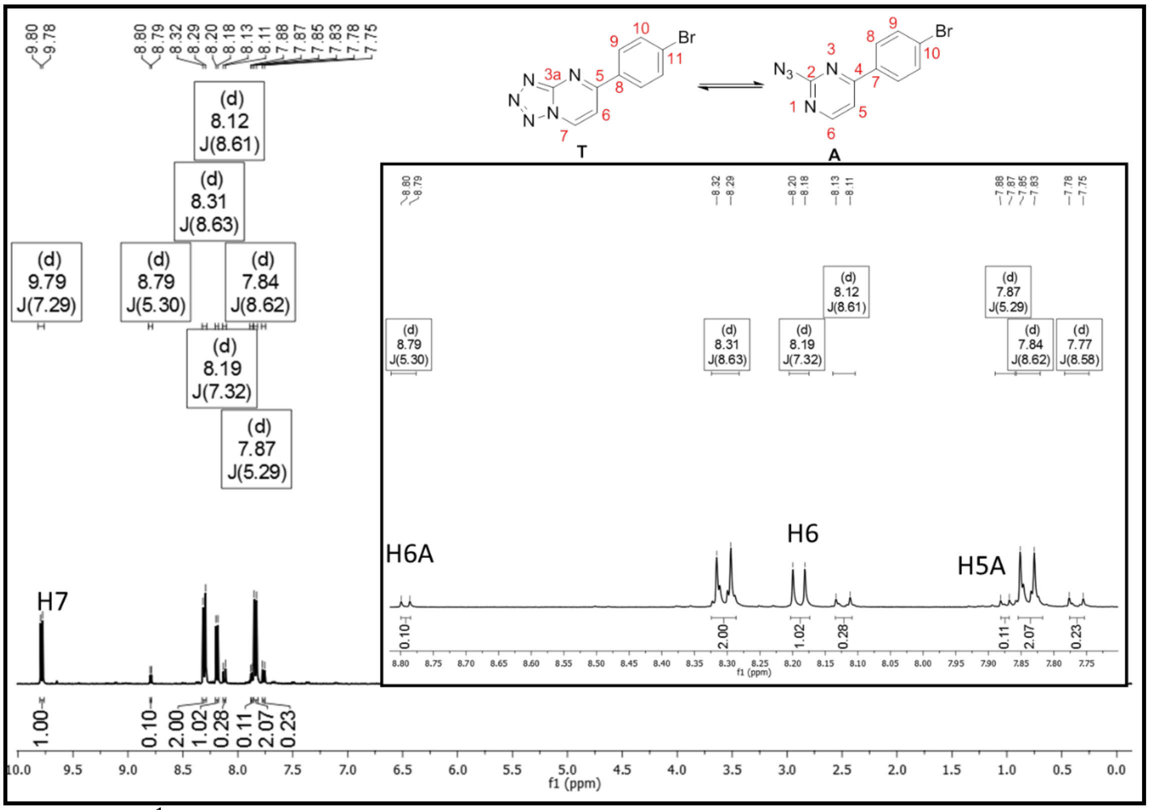

Figure S3: ^{1}HNMR of compound **3d⇌4d** in DMSO-d_6 at 25 °C.

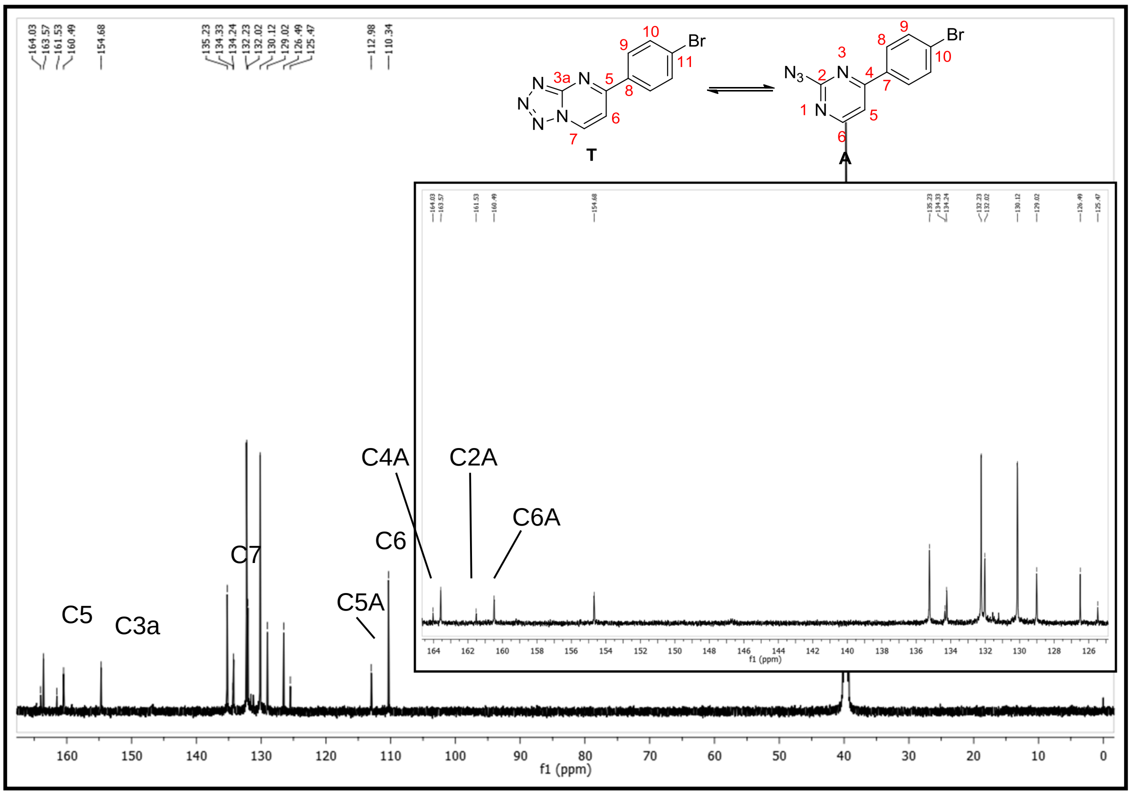

Figure S4: ^{13}C NMR of compound **3d⇌4d** in DMSO-d_6 at 25 °C.

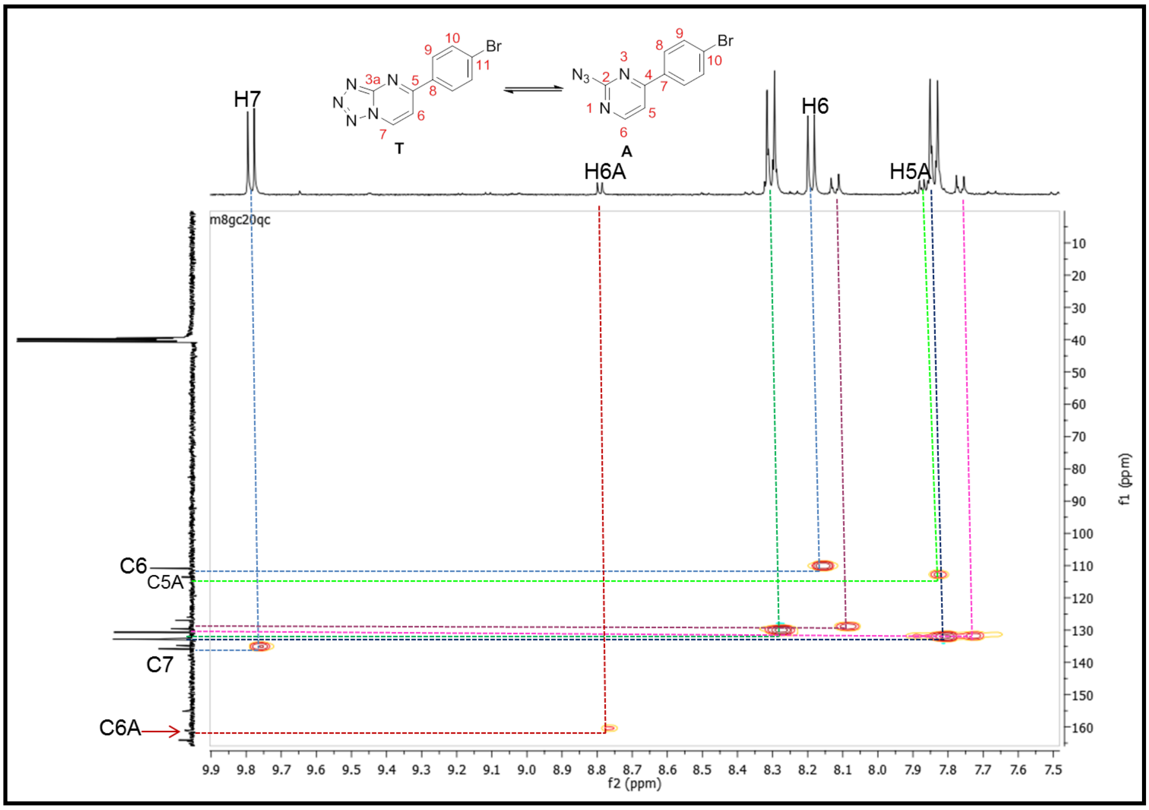

Figure S5: ¹H-¹³C HMQC NMR of compound **3d⇌4d** in DMSO-d_6 at 25 °C.

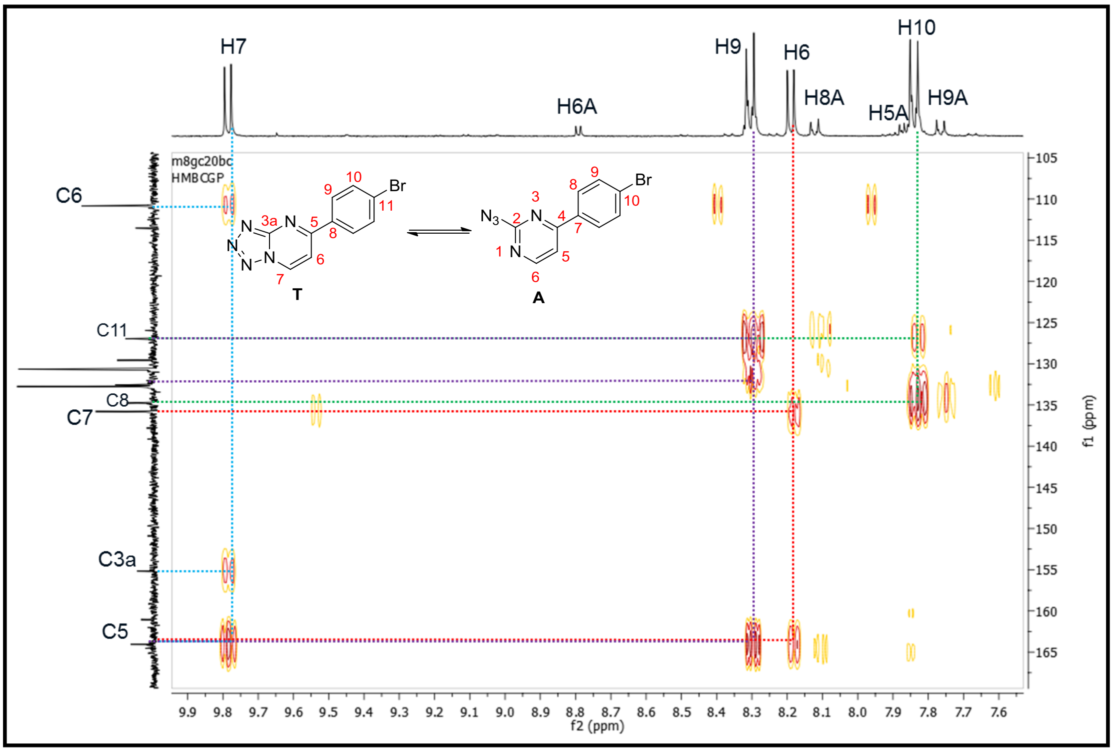

Figure S6: ¹H-¹³C HMBC NMR of compound **3d⇌4d** in DMSO-d_6 at 25 °C.

4. The equilibrium of tetrazolo[1,5-*a*]pyrimidines in solution

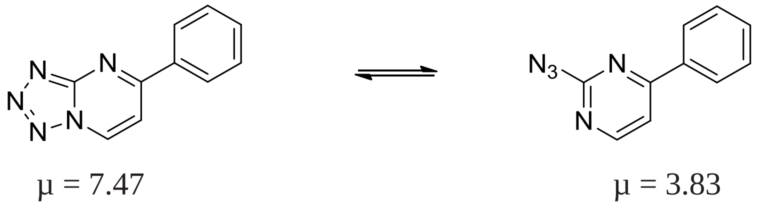

Figure S7: Dipole moment for **3a** and **4a**.

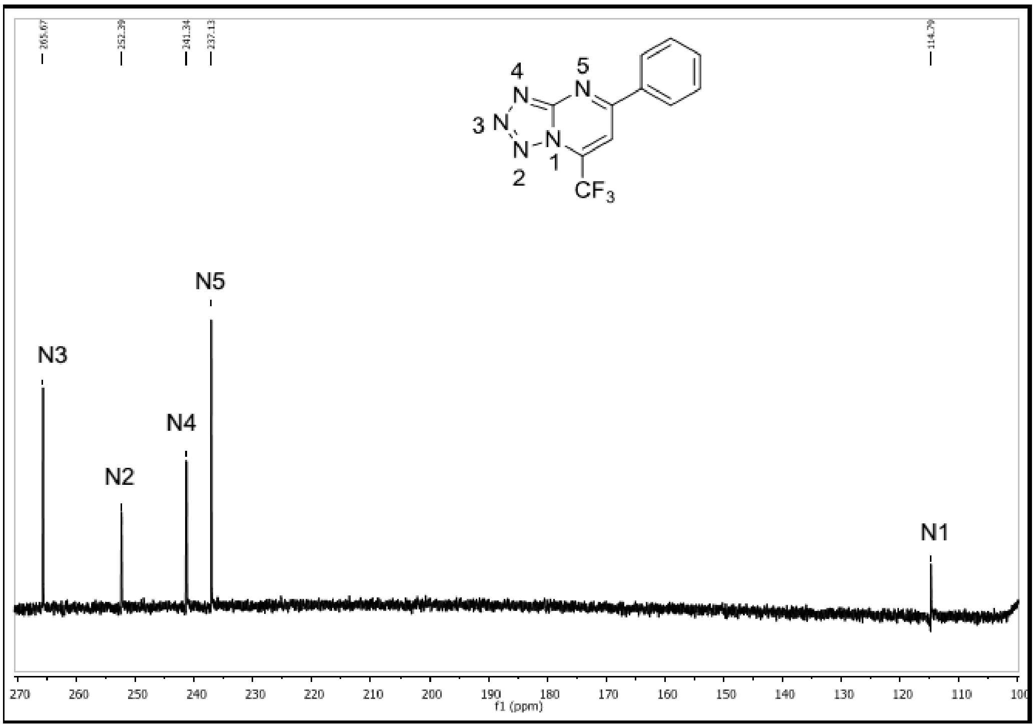

Figure S8: ^{15}N NMR spectra for compound 5-phenyl-7-trifluoromethyltetrazolo[1,5-*a*]pyrimidine (**6a**) in $CDCl_3$.

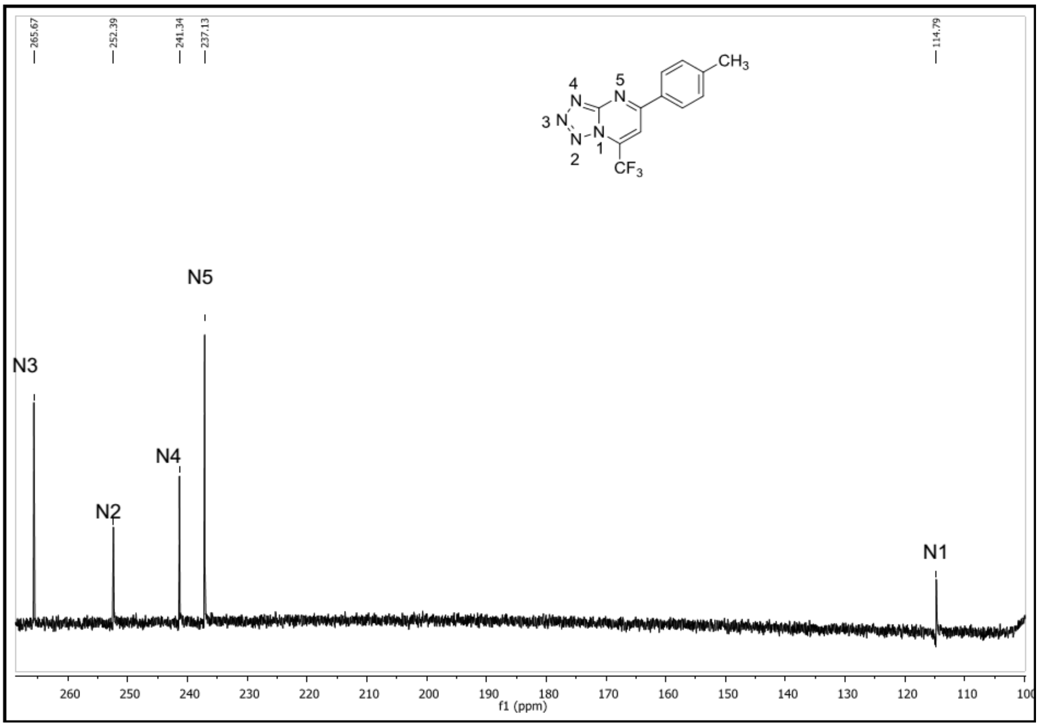

Figure S9: ^{15}N NMR spectra for compound 5-(4-methylphenyl)-7-(trifluoromethyl)tetrazolo[1,5-*a*]pyrimidine (**6g**) in $CDCl_3$.

5. ORTEP and crystallographic data

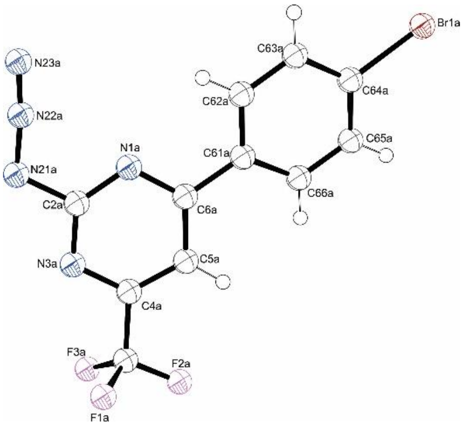

7b

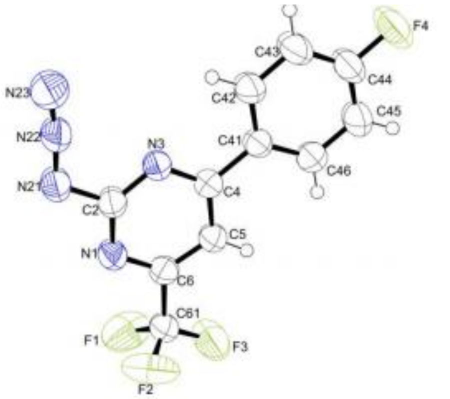

7d

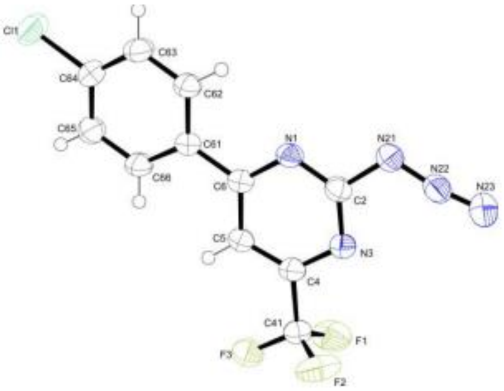

7e

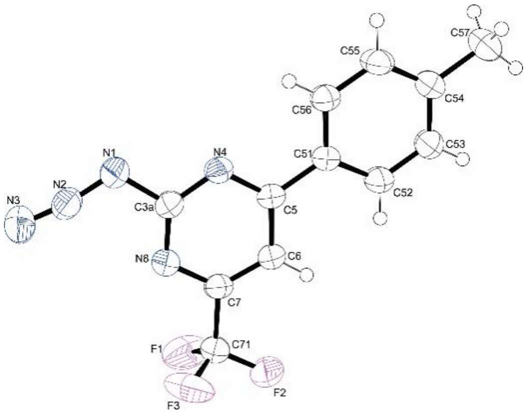

7f

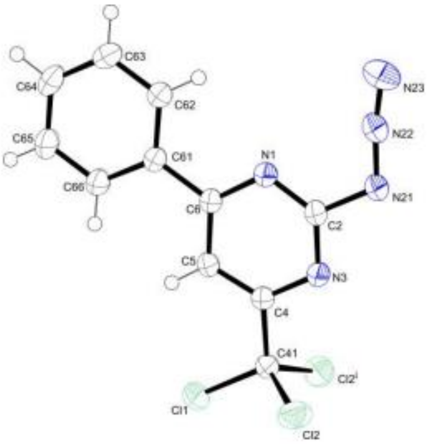

7h

Figure S10: ORTEP® [1] of **7b, 7d, 7e,f, 7h** with thermal ellipsoids drawn at 50% probability level.

Table S1: Crystallographic Data for compounds 7**a**, 7**b**, 7**d** and 7**e**

Compound	**7a**	**7b**	**7d**	**7e**
CCDC	1055527	1055528	1561853	1055526
Empirical formula	$C_{11}H_6F_3N_5$	$C_{11}H_5BrF_3N_5$	$C_{11}H_5F_4N_5$	$C_{11}H_5ClF_3N_5$
Molecular weight	265.21	344.11	283.20	299.65
Temperature (K)	293 (2)	293 (2)	293 (2)	293 (2)
Crystal system	Monoclinic	Triclinic	Triclinic	Monoclinic
Space group	C2/*c*	P-1	P-1	C2/*c*
Cell parameters				
a (Å)	9.6721(3)	8.1168(4)	7.7390(4)	21.9971(7)
b (Å)	18.4409(7)	11.9327(7)	11.7442(6)	4.9657(2)
c (Å)	13.2081(4)	15.2077(9)	14.0976(7)	22.1893(8)
α (°)	90	67.481(3)	73.475(3)	90
β (°)	103.411(2)	87.182(3)	83.479(3)	99.812(2)
γ (°)	90	71.989(3)	76.629(3)	90
V ($Å^3$)	2291.59 (13)	1289.94	1193.52	2388.30
Z	8	4	4	8
Calcd. density ($g.cm^{-3}$)	1.537	1.772	1.576	1.667
Abs. Coef. (mm^{-1})	0.134	3.219	0.145	0.355
F (000)	1072	672	568	1200
Crystal size (mm)	0.44 x 0.35 x 0.25	0.68 x 0.13 x 0.12	0.65 x 0.63 x 0.39	0.58 x 0.39 x 0.32
θ data collection (deg)	2.21 to 28.37	1.45 to 27.85	1.85 to 27.28	1.86 to 27.17
h,k,l range	-12≤h≤12, -19≤k≤24, -17≤l≤17	-10≤h≤10, -15≤k≤15, -19≤l≤19	-9≤h≤9, -15≤k≤15, -18≤l≤18	-28≤h≤28, -6≤k≤6, -28≤l≤28
Reflections collected/unique	33551 / 2868 [R(int) = 0.0263]	39166 / 5980 [R(int) = 0.0368]	28216 / 5315 [R(int) = 0.0239]	16975 / 2659 [R(int) = 0.0214]
Data/restraints/parameters	2868 / 0 / 172	5980 / 0 / 361	5315 / 0 / 361	2659 / 0 / 181
Absorption correction	Gaussian	Gaussian	Gaussian	Gaussian
Refinement method	Full-matrix least-squares on F^2	Full-matrix least-squares on F^2	Full-matrix least-squares on F^2	Full-matrix least-squares on F^2
Final *R* indices	R1 = 0.0500, wR2 = 0.1362	R1 = 0.0534, wR2 = 0.1475	R1 = 0.0588, wR2 = 0.1754	R1 = 0.0373, wR2 = 0.0948
R all data	R1 = 0.0657, wR2 = 0.1484	R1 = 0.0997, wR2 = 0.1708	R1 = 0.0786, wR2 = 0.2035	R1 = 0.0451, wR2 = 0.1005
Goodness of fit on F^2	1.066	1.072	0.790	1.051
Largest diff. peak and hole($eÅ^3$)	0.467 and -0.334	0.977 and -0.811	0.671 and -0.399	0.292 and -0.262

Table S2: Crystallographic Data for compounds **7f**, 7**h** and **8i**

Compound	**7f**	**7h**	**8i**
CCDC	1055530	1055529	1042114
Empirical formula	$C_{12}H_8F_3N_5$	$C_{11}H_6Cl_3N_5$	$C_6H_4Cl_3N_5$
Molecular weight	279.23	314.56	252.49
Temperature (K)	293	100	100
Crystal system	Monoclinic	Orthorhombic	Monoclinic
Space group	C2/*c*	Pnma	$P2_1/c$
Cell parameters			
a (Å)	21.9497(14)	18.9696(8)	8.2837(3)
b (Å)	4.9922(4)	6.7126(3)	8.9787(3)
c (Å)	22.4848(16)	10.0801(4)	13.4877(4)
α (°)	90 deg.	90.	90
β (°)	100.799(4)	90	105.6690(10)
γ (°)	90	90	90
V ($Å^3$)	2420.2	1283.55	965.89
Z	8	4	4
Calcd. density ($g.cm^{-3}$)	1.533	1.628	1.736
Abs. Coef. (mm^{-1})	0.131	0.705	0.913
F (000)	1136	632	504
Crystal size (mm)	0.83 x 0.40 x 0.32	0.72 x 0.49 x 0.24	0.62 x 0.49 x 0.46
θ data collection (deg)	1.84 to 28.35	2.15 to 27.21	2.55 to 27.10
h,k,l range	-28≤h≤28,<br>-4≤k≤6,<br>-30≤l≤29	-24≤h≤24,<br>-8≤k≤8,<br>-12≤l≤12	-6≤h≤10,<br>-11≤k≤9,<br>-16≤l≤14
Reflections collected/unique	18808 / 3016 [R(int) = 0.0313]	18393 / 1551 [R(int) = 0.0188]	13530 / 2028 [R(int) = 0.0189]
Data/restraints/parameters	3016 / 0 / 181	1551 / 0 / 112	2028 / 0 / 127
Absorption correction	Gaussian	Gaussian	Gaussian
Refinement method	Full-matrix least-squares on F^2	Full-matrix least-squares on F^2	Full-matrix least-squares on F^2
Final *R* indices	R1 = 0.0506,<br>wR2 = 0.1416	R1 = 0.0277,<br>wR2 = 0.0771	R1 = 0.0420,<br>wR2 = 0.1086
R all data	R1 = 0.0702,<br>wR2 = 0.1558	R1 = 0.0300,<br>wR2 = 0.0793	R1 = 0.0499,<br>wR2 = 0.1160
Goodness of fit on F^2	1.052	1.080	1.065
Largest diff. peak and hole($eÅ^3$)	0.454 and -0.253	0.348 and -0.223	0.509 and -0.462

6. HOMO coefficients

Table S3: HOMO coefficients for the aminotetrazole nucleophile.

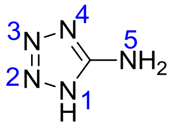	**1**	**2**	**3**	**4**	**5**
	0.077	0.193	0.094	0.182	0.232

7. FTIR spectrum of compounds **7a**,**b** and 7**f**

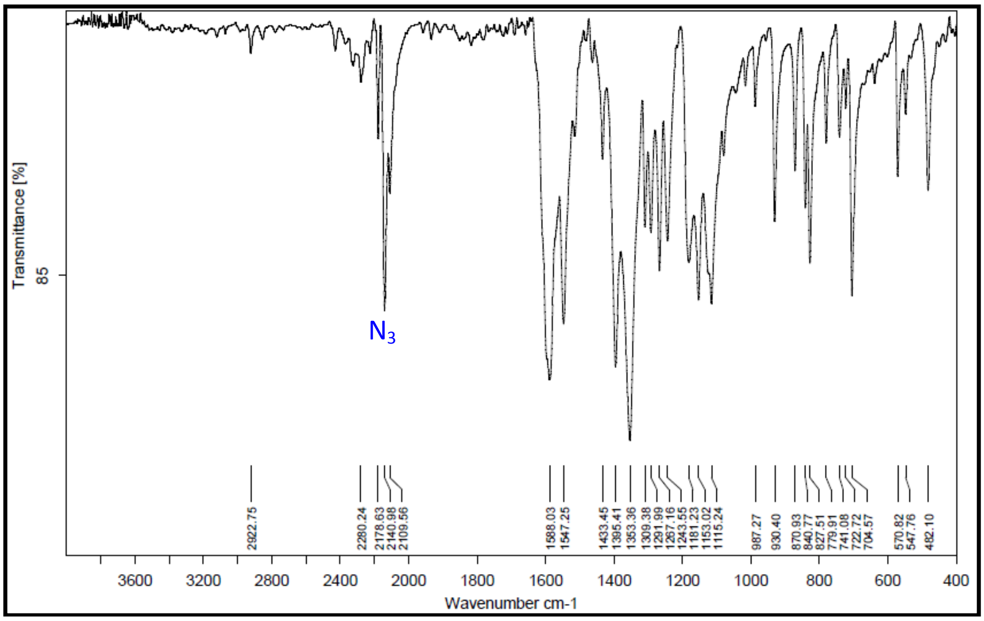

Figure S11: FTIR spectra of compound **7a** using KBr pellet.

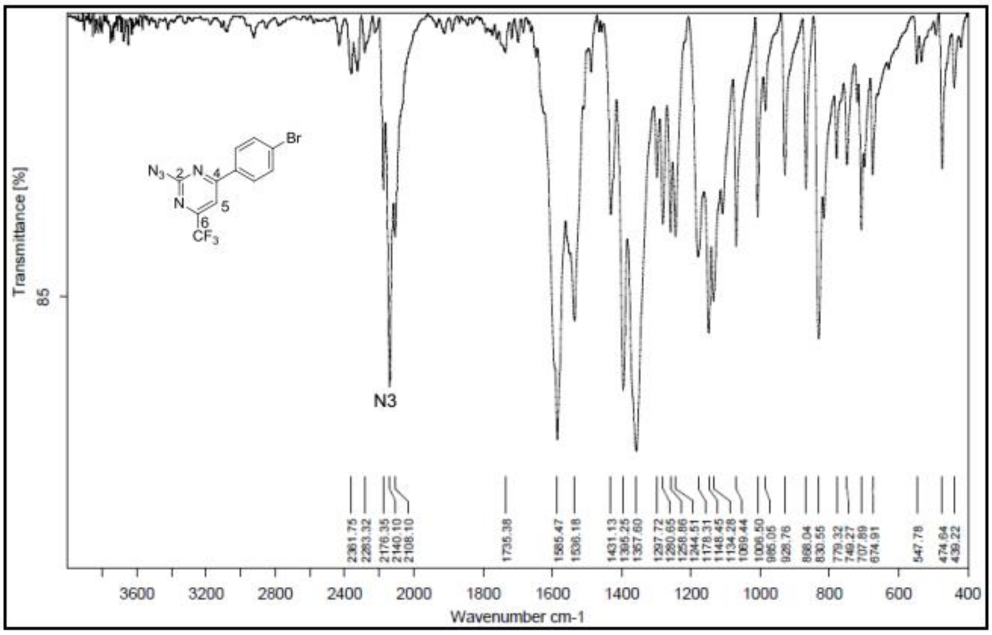

Figure S12: FTIR spectra of compound **7b** using KBr pellet.

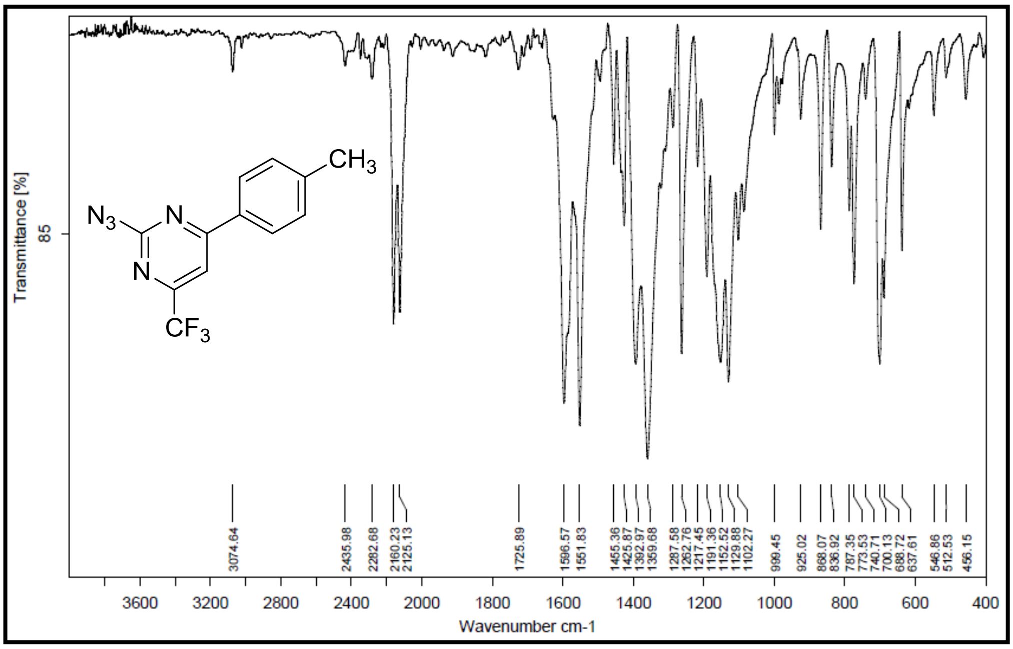

Figure S13: FTIR spectra of compound **7f** using KBr pellet.

8. Mechanism of trifluoromethylated tetrazolo[1,5-*a*]pyrimidines (**6a–i**) formation.

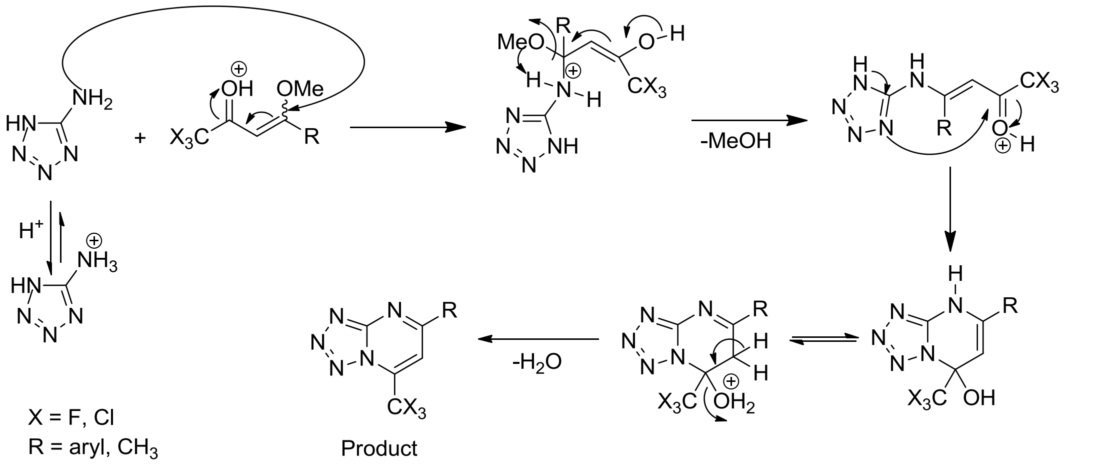

Figure S14: Scheme that demonstrate the mechanism of trifluoromethylated tetrazolo[1,5-*a*]pyrimidines (**6a–i**) formation.

9. NMR spectrum of compound **3b–f**, **5i**, **6h–i** and **8a–c**

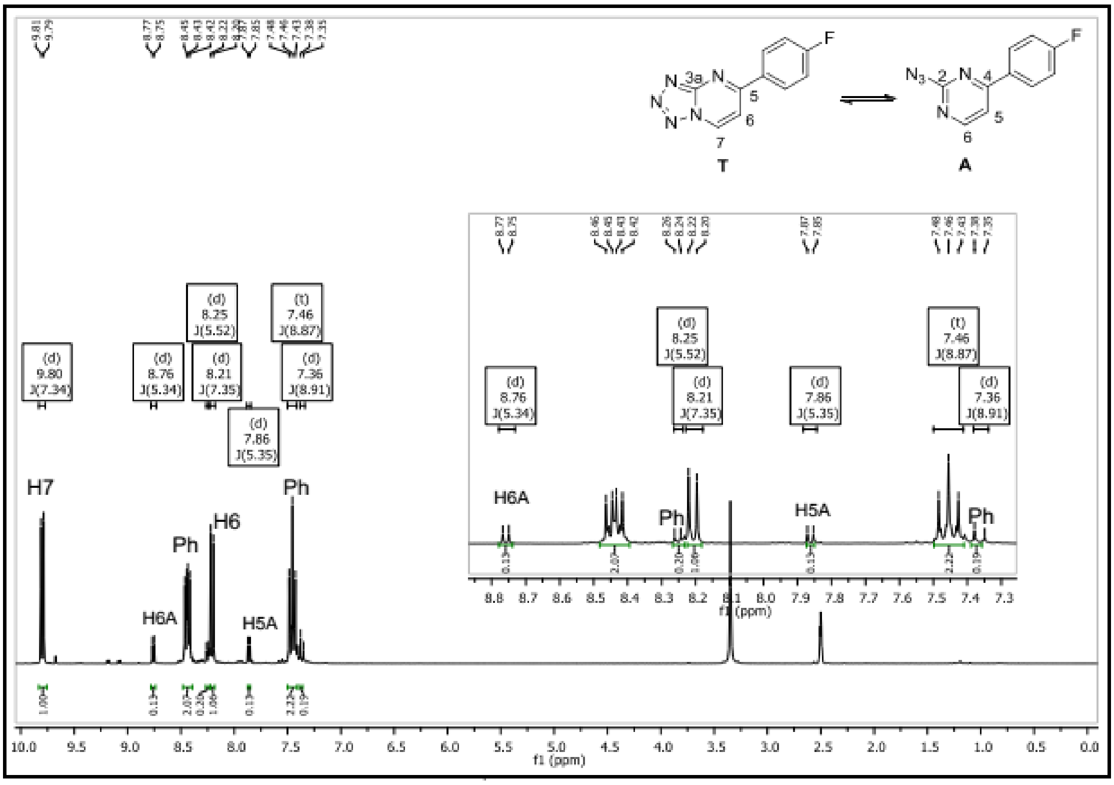

Figure S15: ^{1}H NMR of compound **3b** in DMSO-d_6.

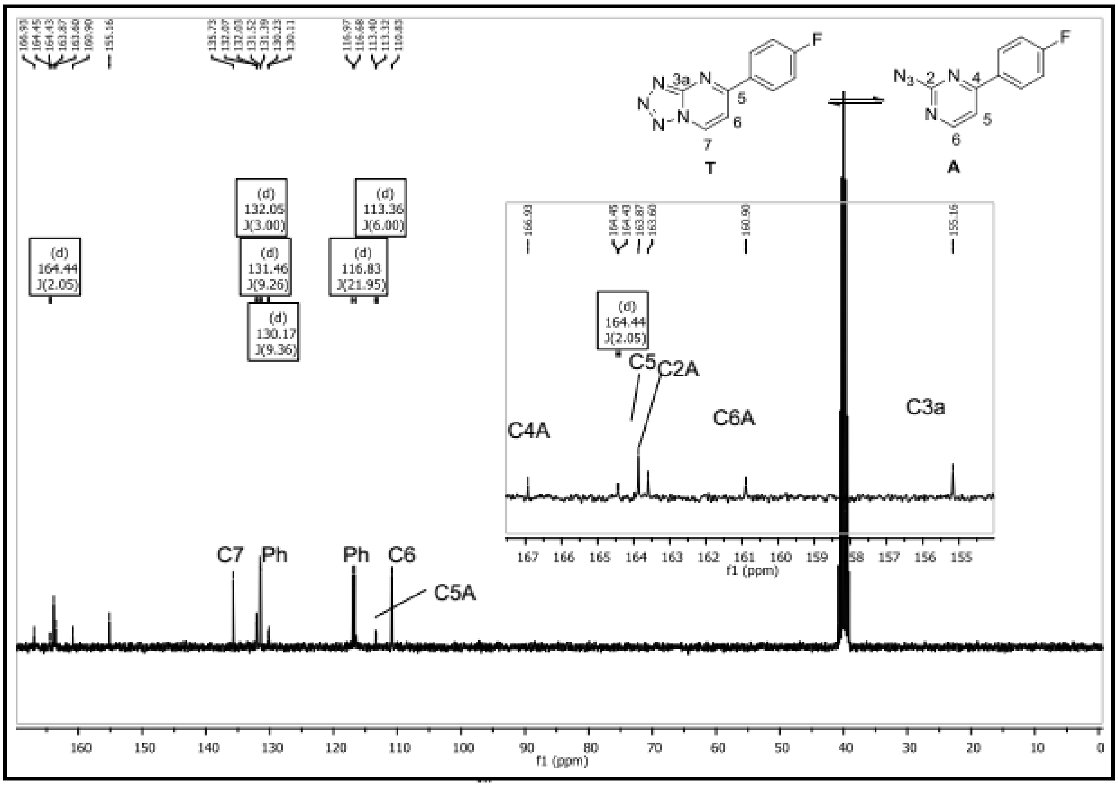

Figure S16: ^{13}C NMR of compound **3b** in DMSO-d_6.

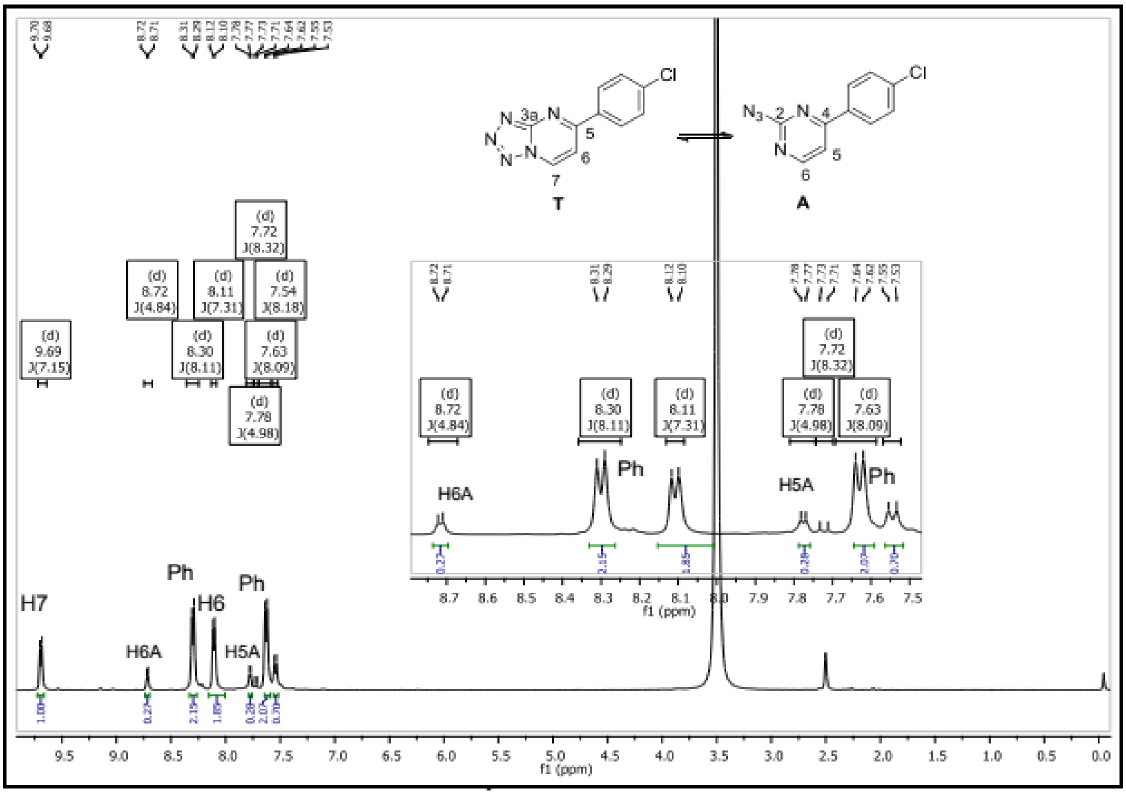

Figure S17: ^{1}H NMR of compound **3c** in DMSO-d_6.

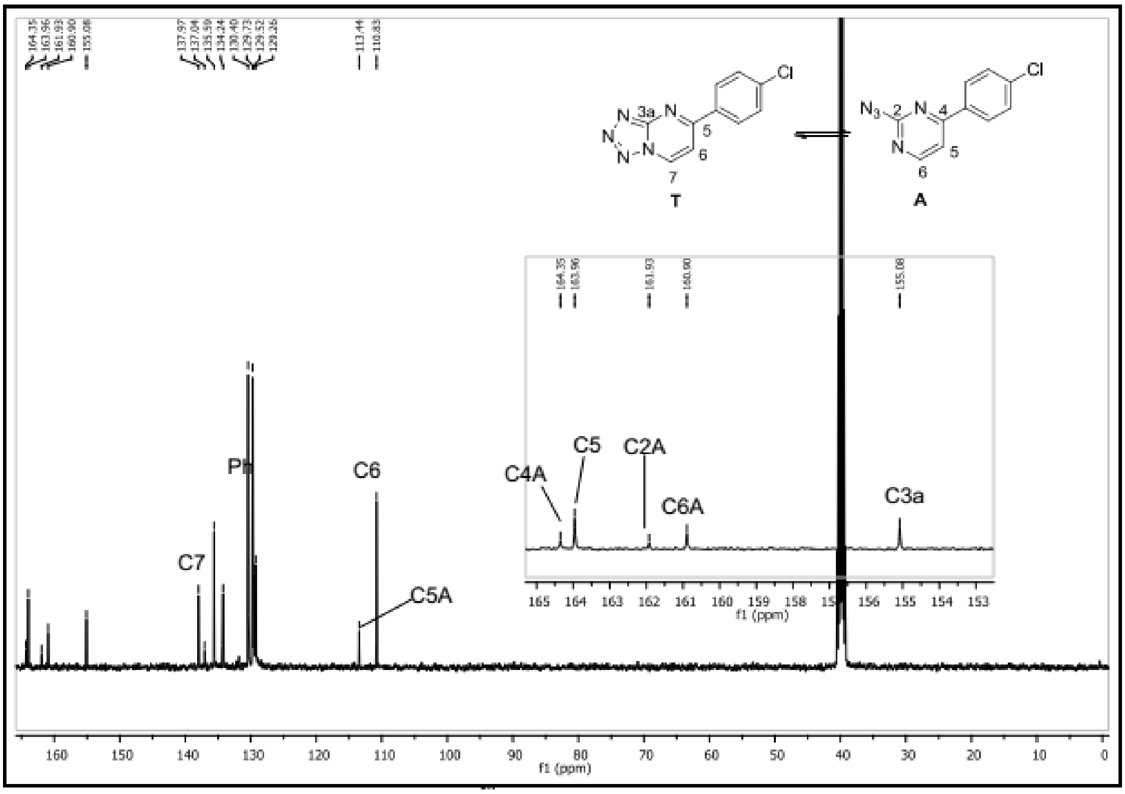

Figure S18: ^{13}C NMR of compound **3c** in DMSO-d_6.

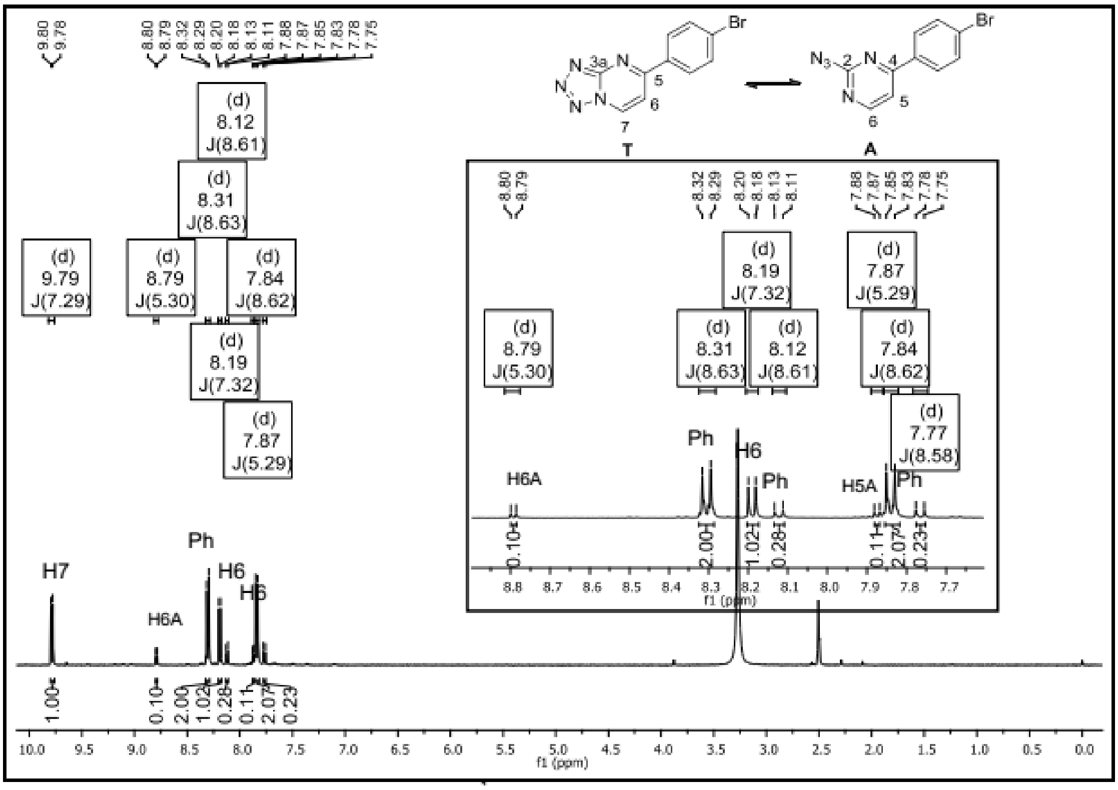

Figure S19: ^{1}H NMR of compound **3d** in DMSO-d_6.

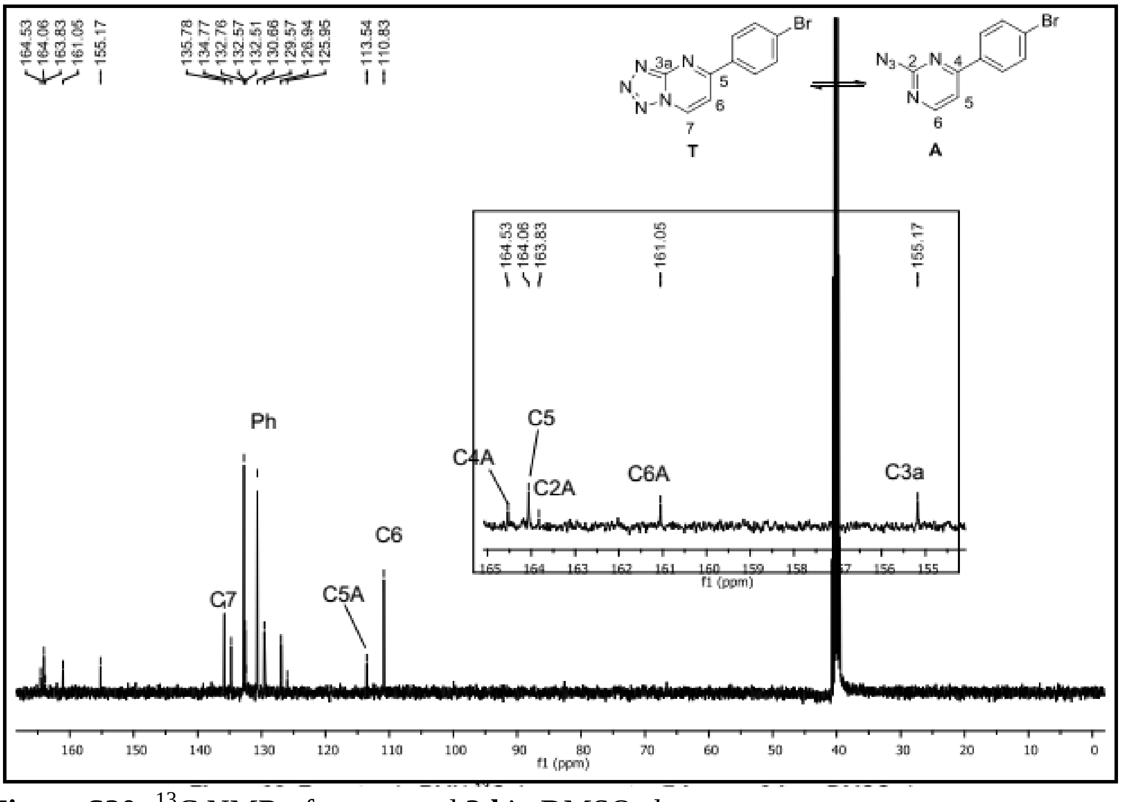

Figure S20: ^{13}C NMR of compound **3d** in DMSO-d_6.

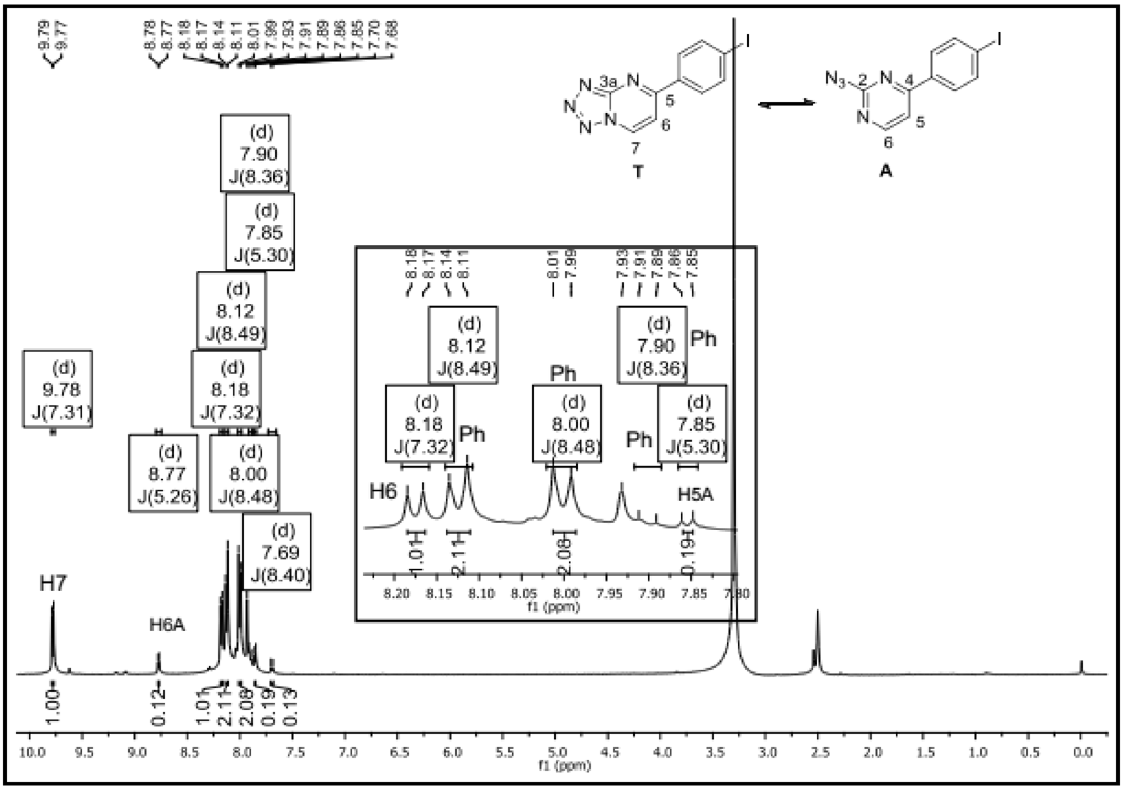

Figure S21: ^{1}H NMR of compound **3e** in DMSO-d_6.

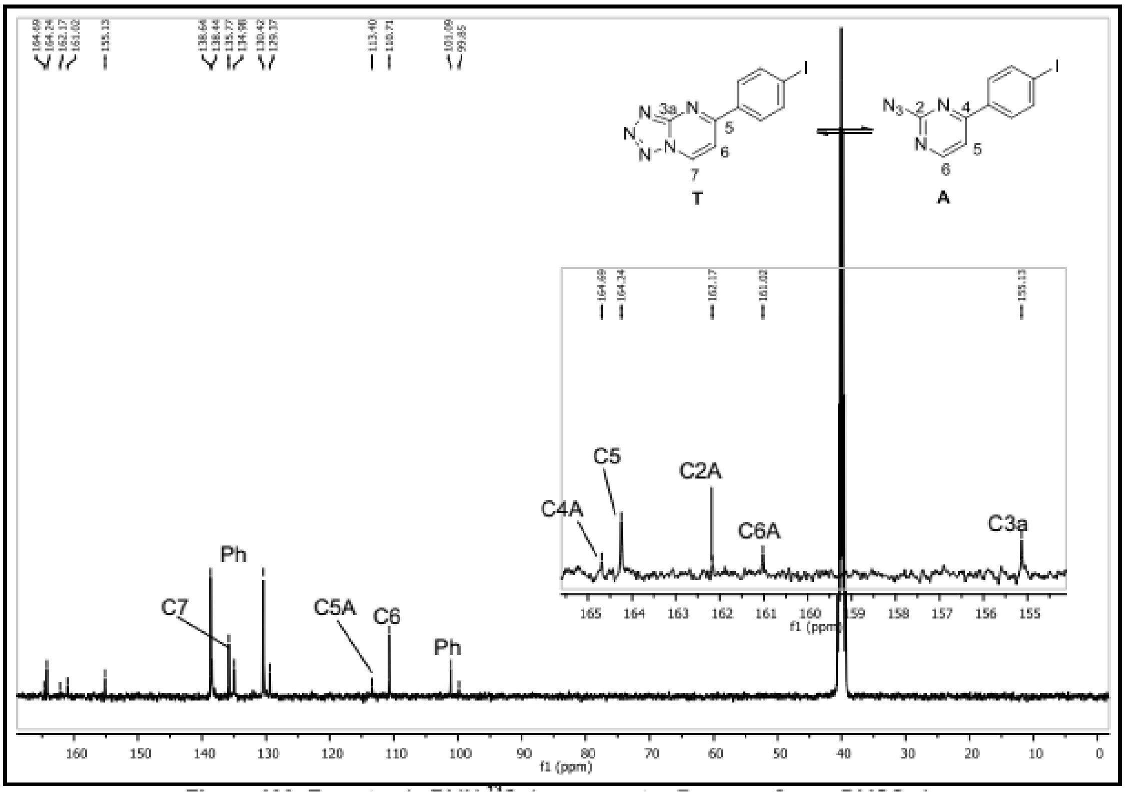

Figure S22: ^{13}C NMR of compound **3e** in DMSO-d_6.

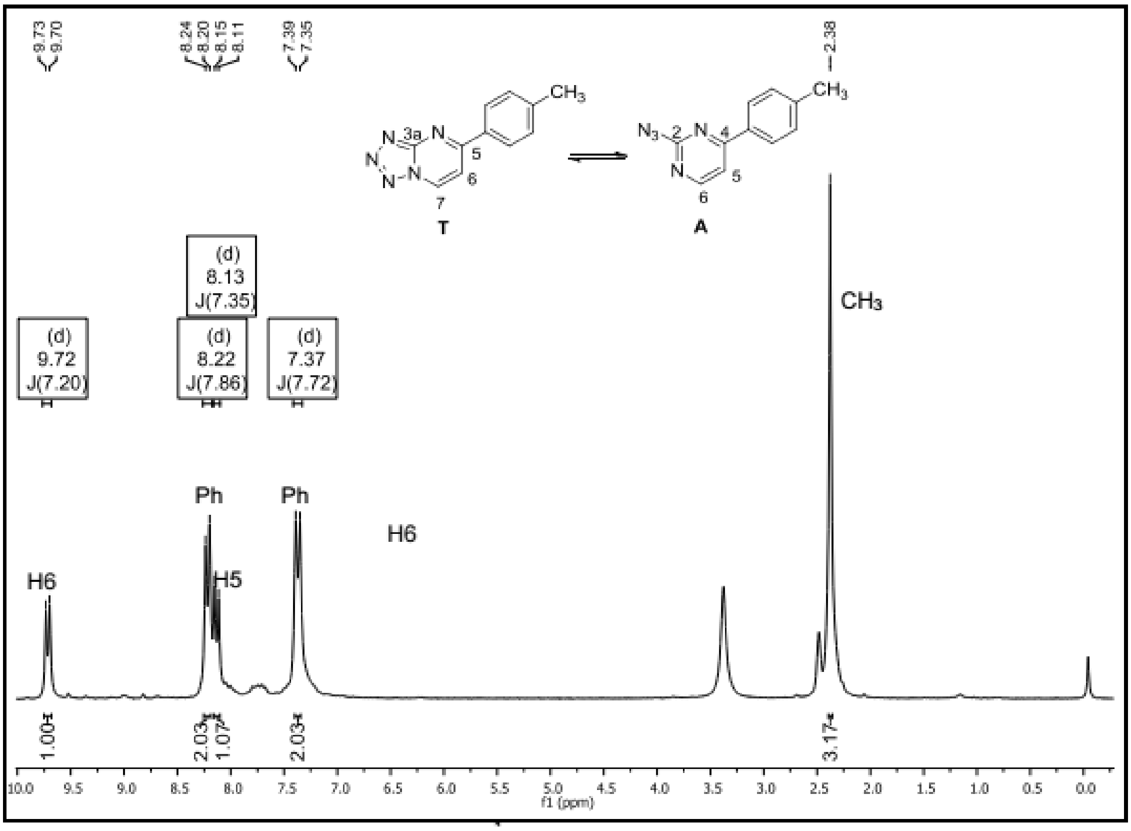

Figure S23: ^{1}H NMR of compound **3f** in DMSO-d_6.

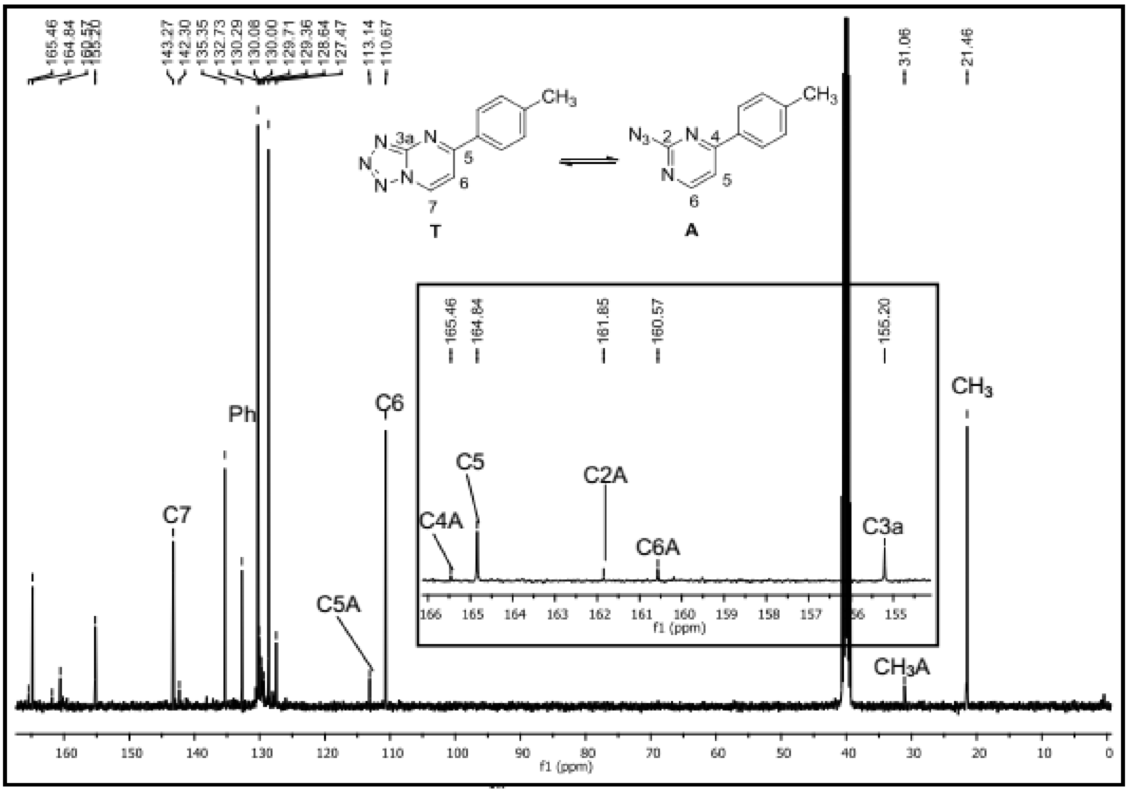

Figure S24: ^{13}C NMR of compound **3f** in DMSO-d_6.

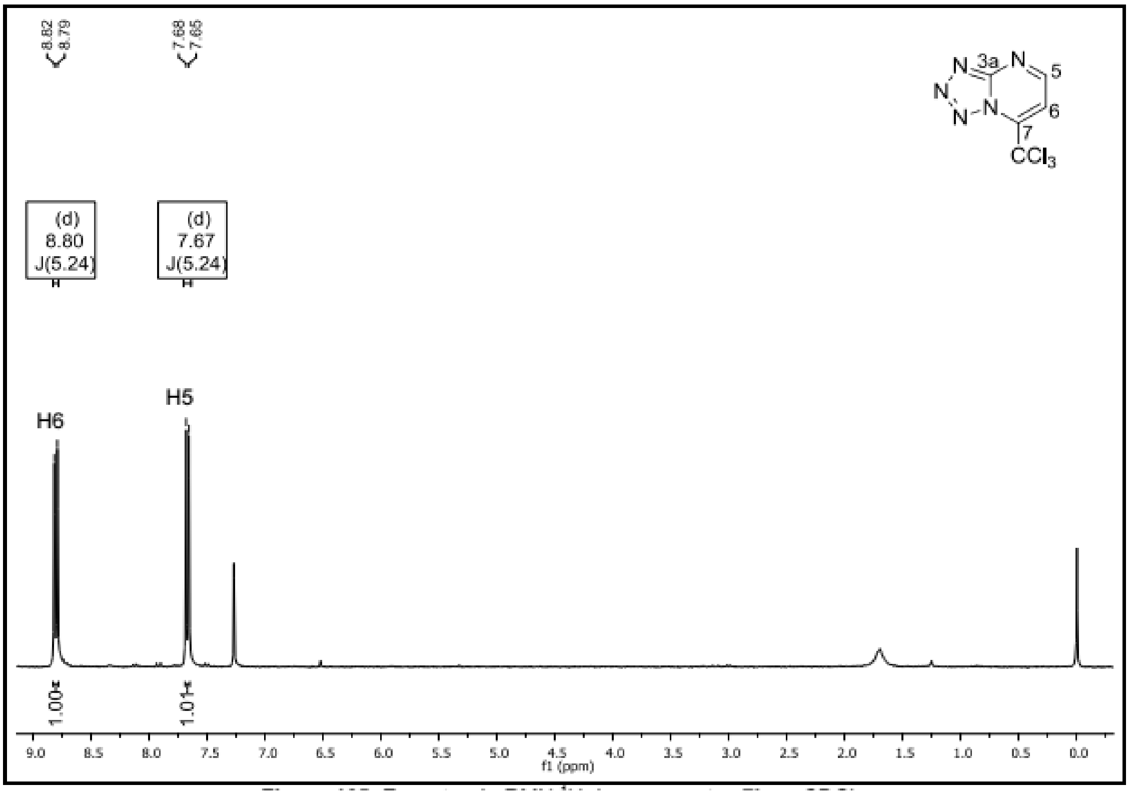

Figure S25: ^{1}H NMR of compound **5i** in $CDCl_3$.

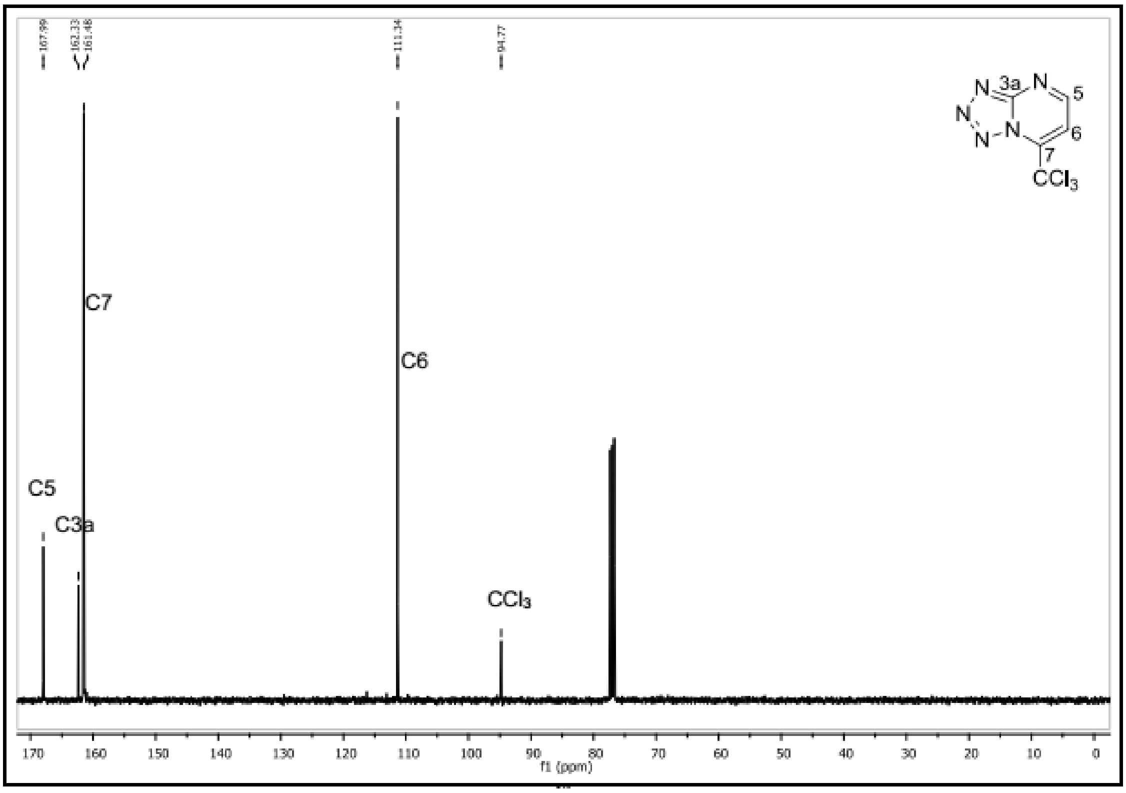

Figure S26: ^{13}C NMR of compound **5i** in $CDCl_3$.

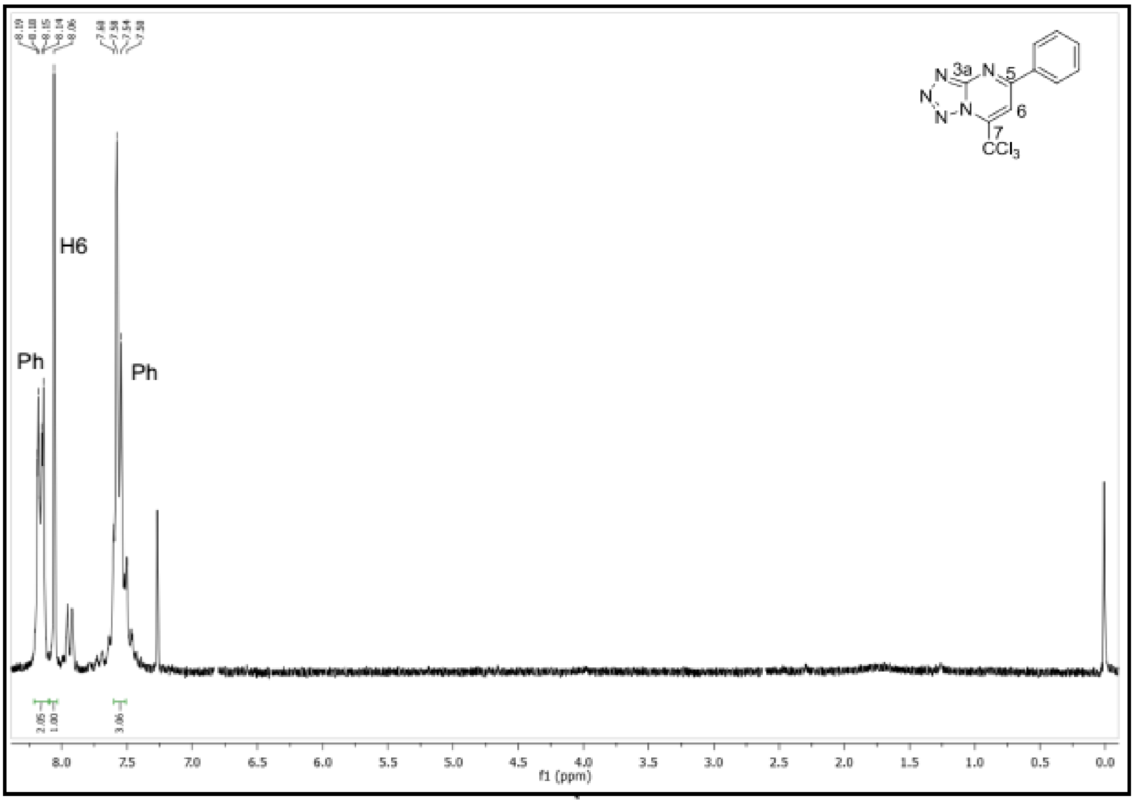

Figure S27: ^{1}H NMR of compound **6h** in $CDCl_3$.

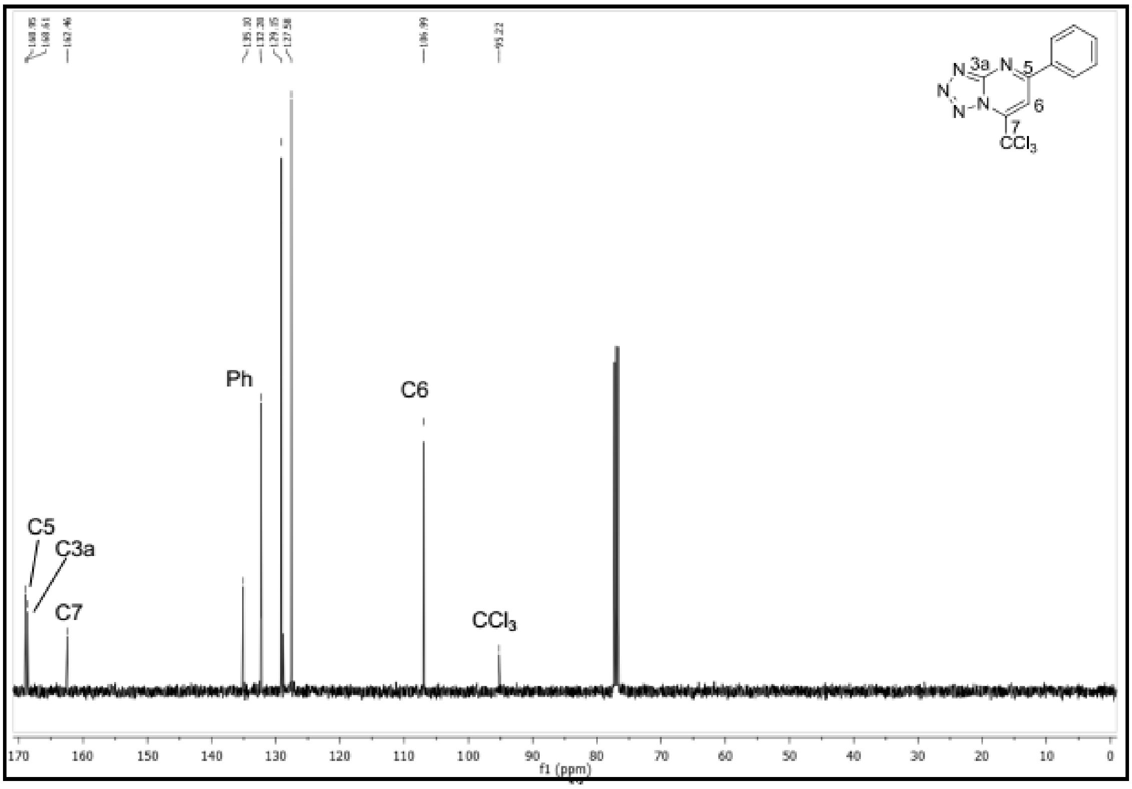

Figure S28: ^{13}C NMR of compound **6h** in $CDCl_3$.

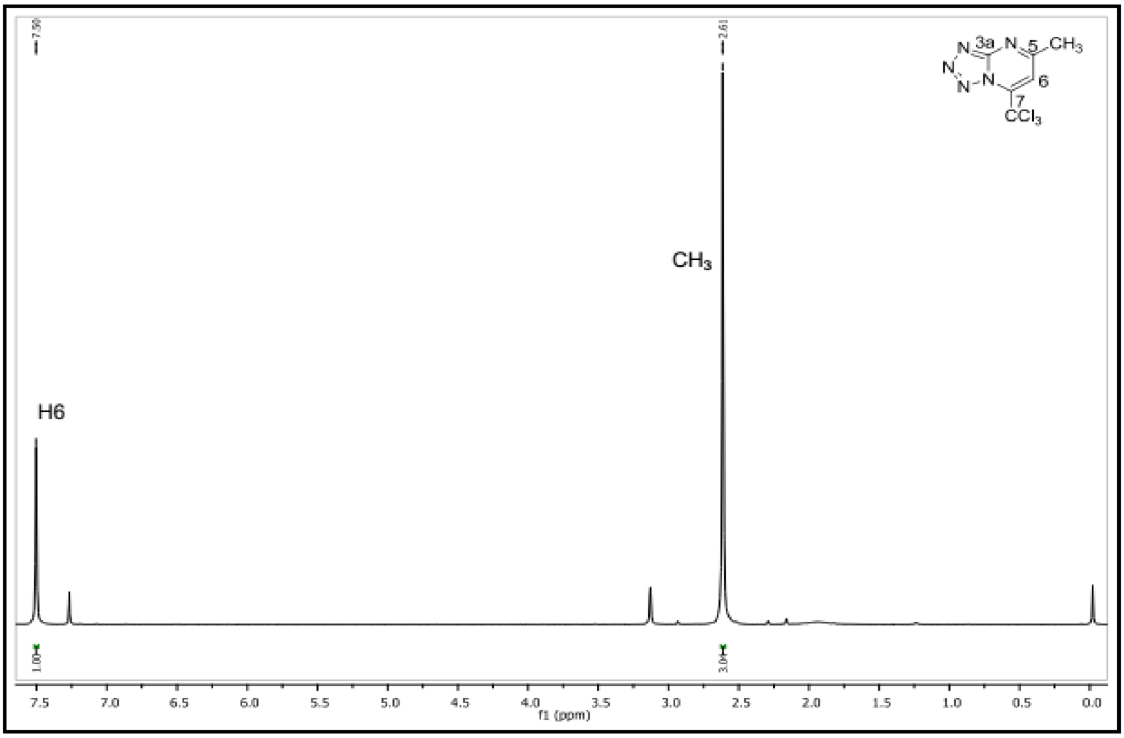

Figure S29: ^{1}H NMR of compound **6i** in $CDCl_3$.

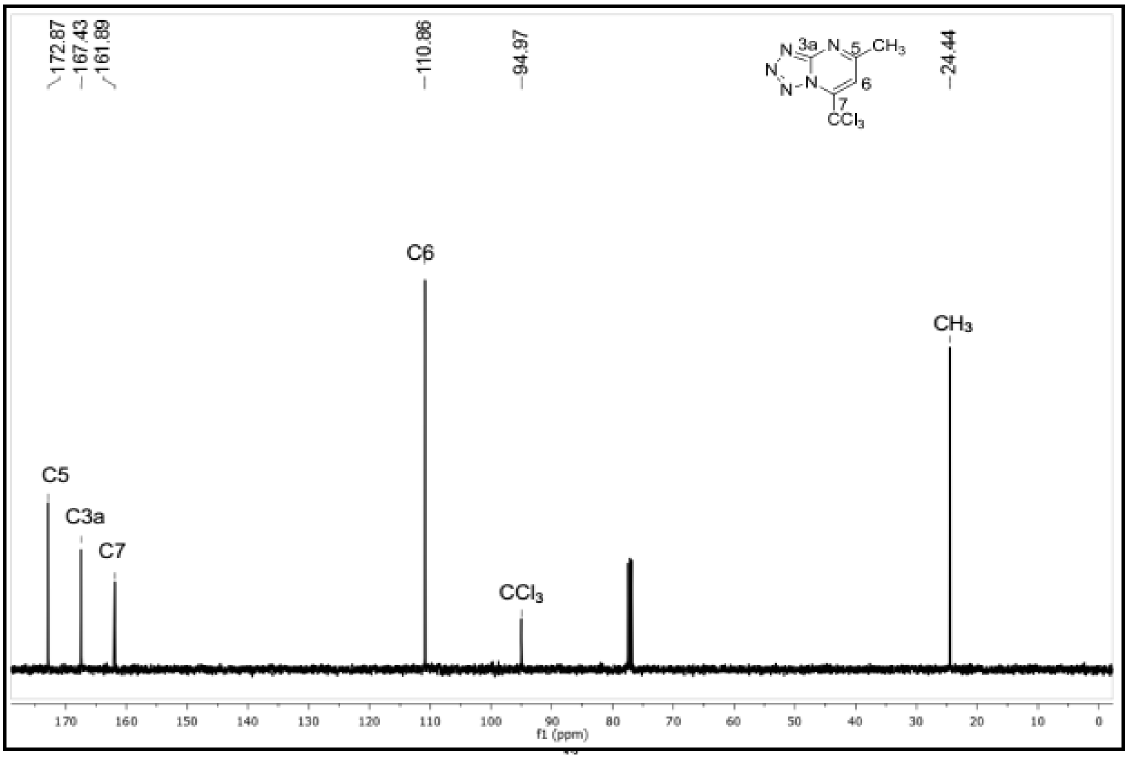

Figure S30: ^{13}C NMR of compound **6i** in $CDCl_3$.

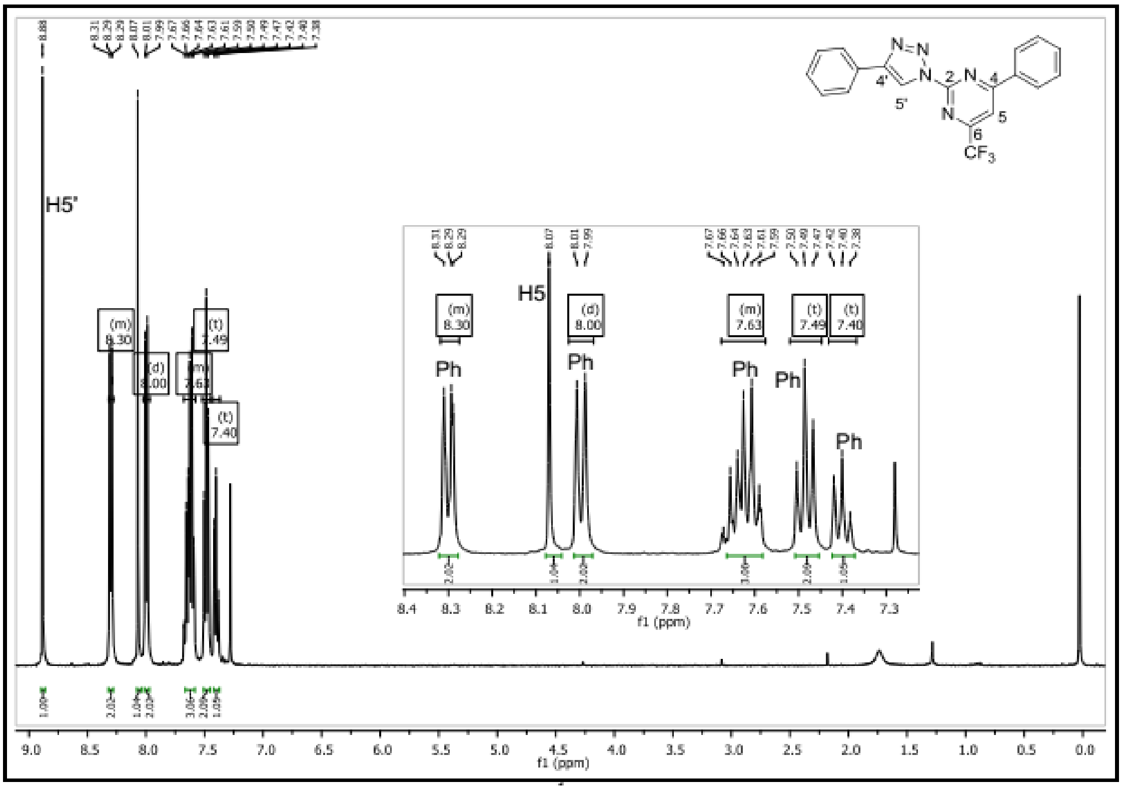

Figure S31: ^{1}H NMR of compound **8a** in $CDCl_3$.

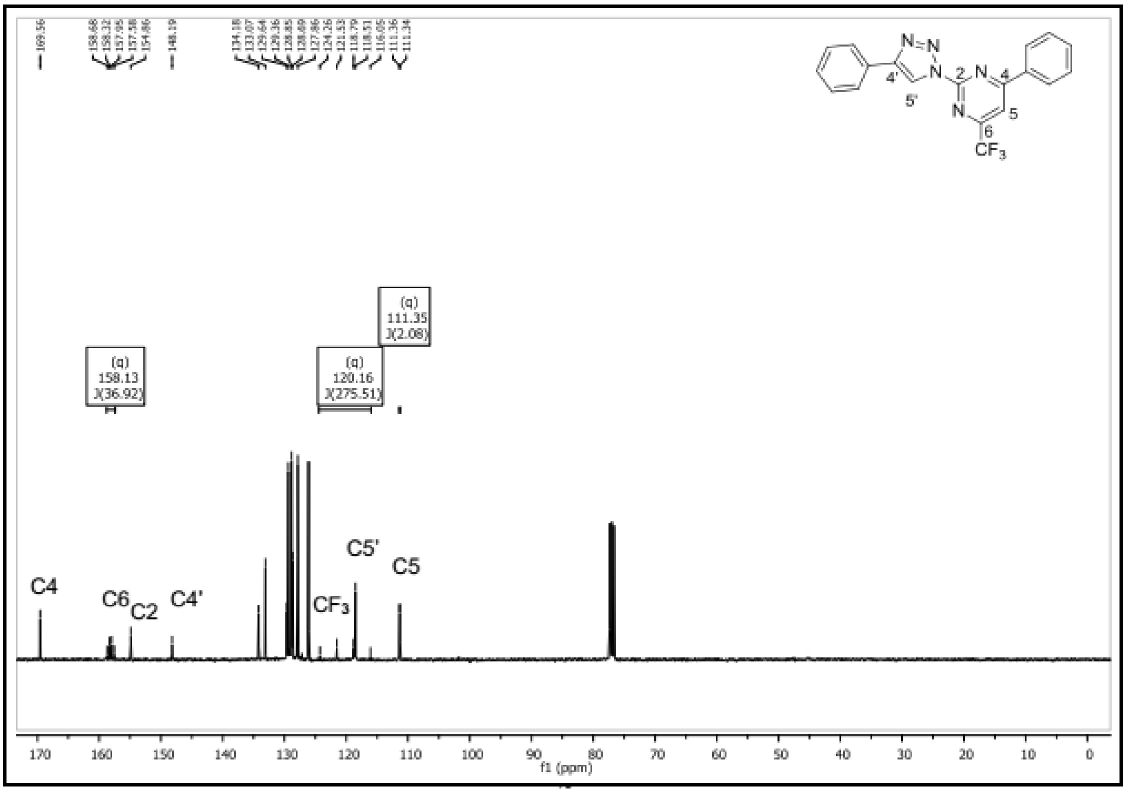

Figure S32: ^{13}C NMR of compound **8a** in $CDCl_3$.

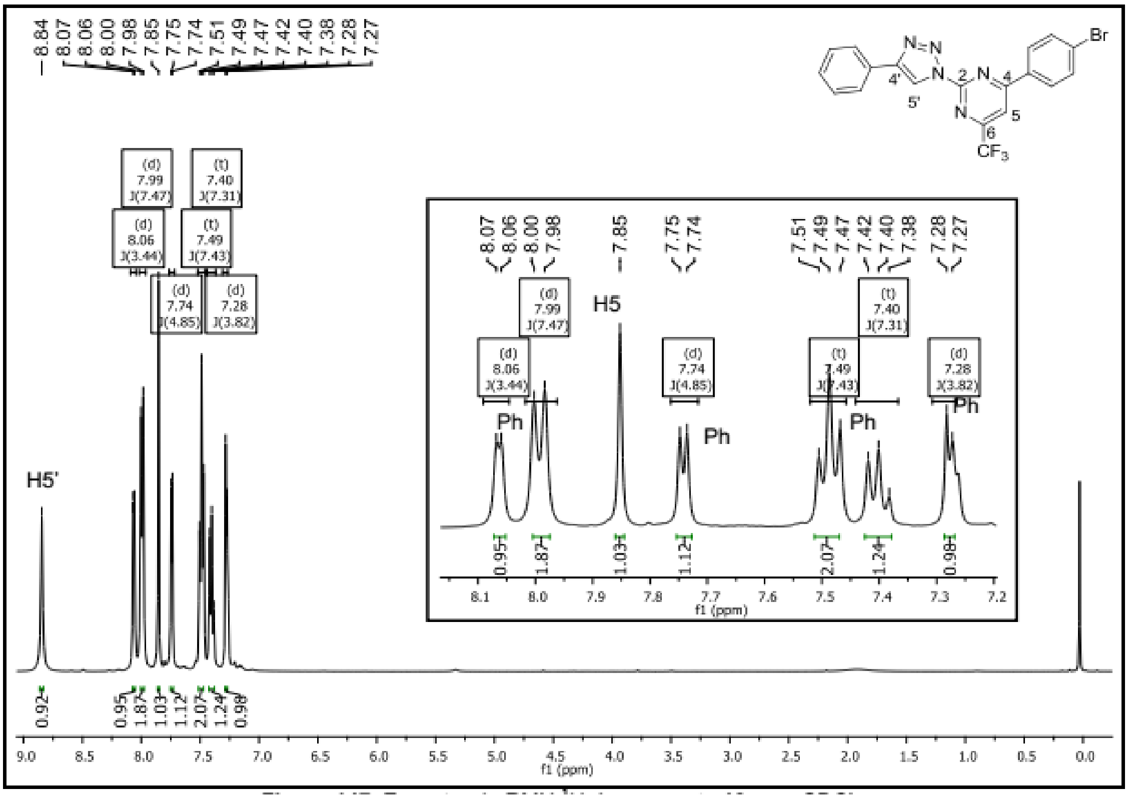

Figure S33: ^{1}H NMR of compound **8b** in $CDCl_3$.

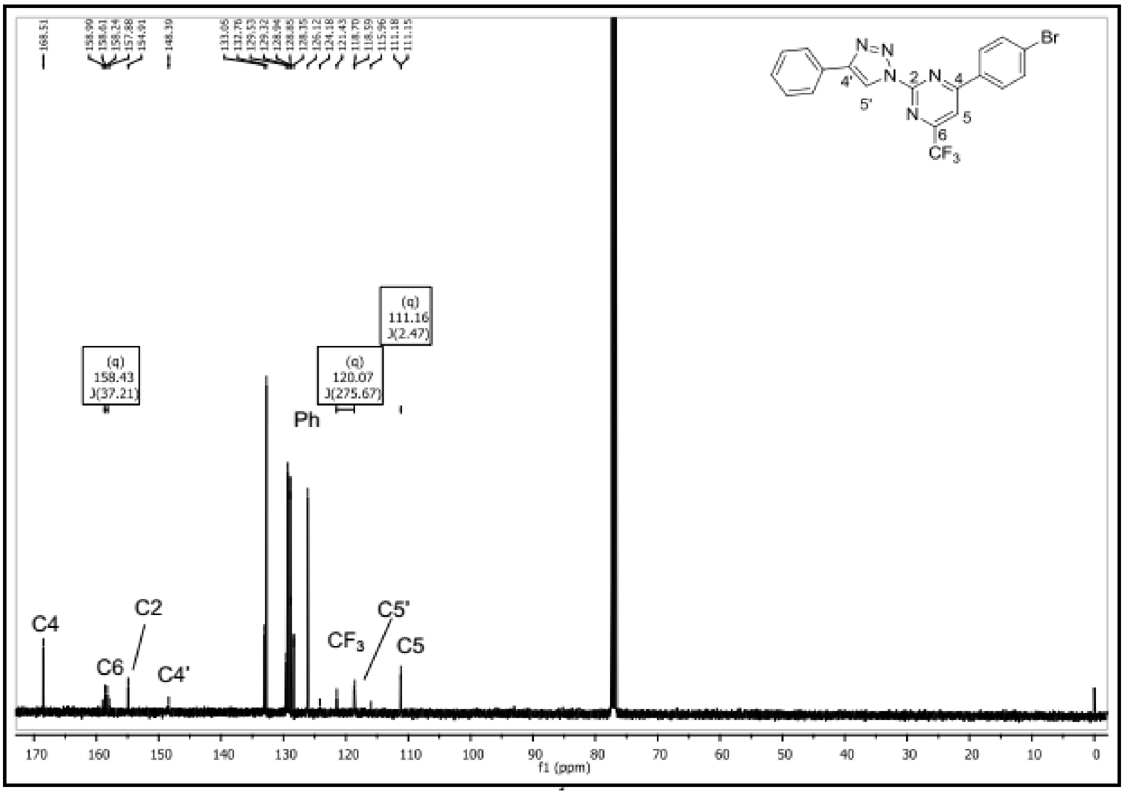

Figure S34: ^{13}C NMR of compound **8b** in $CDCl_3$.

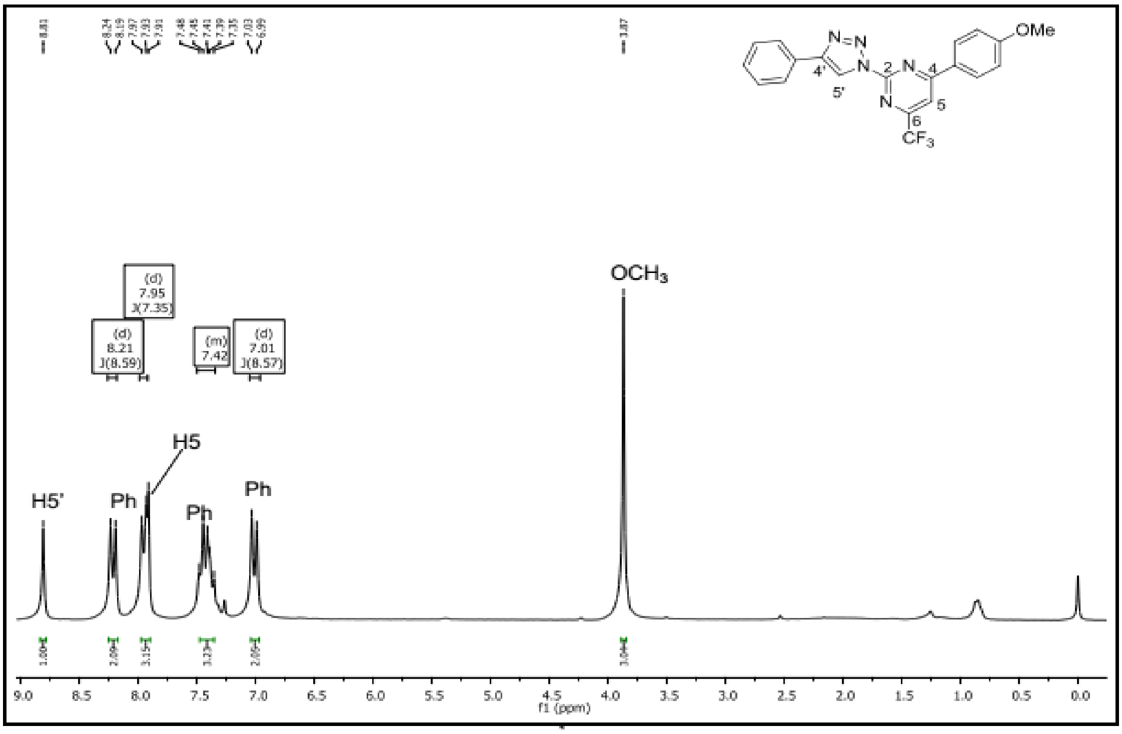

Figure S35: ^{1}H NMR of compound **8c** in $CDCl_3$.

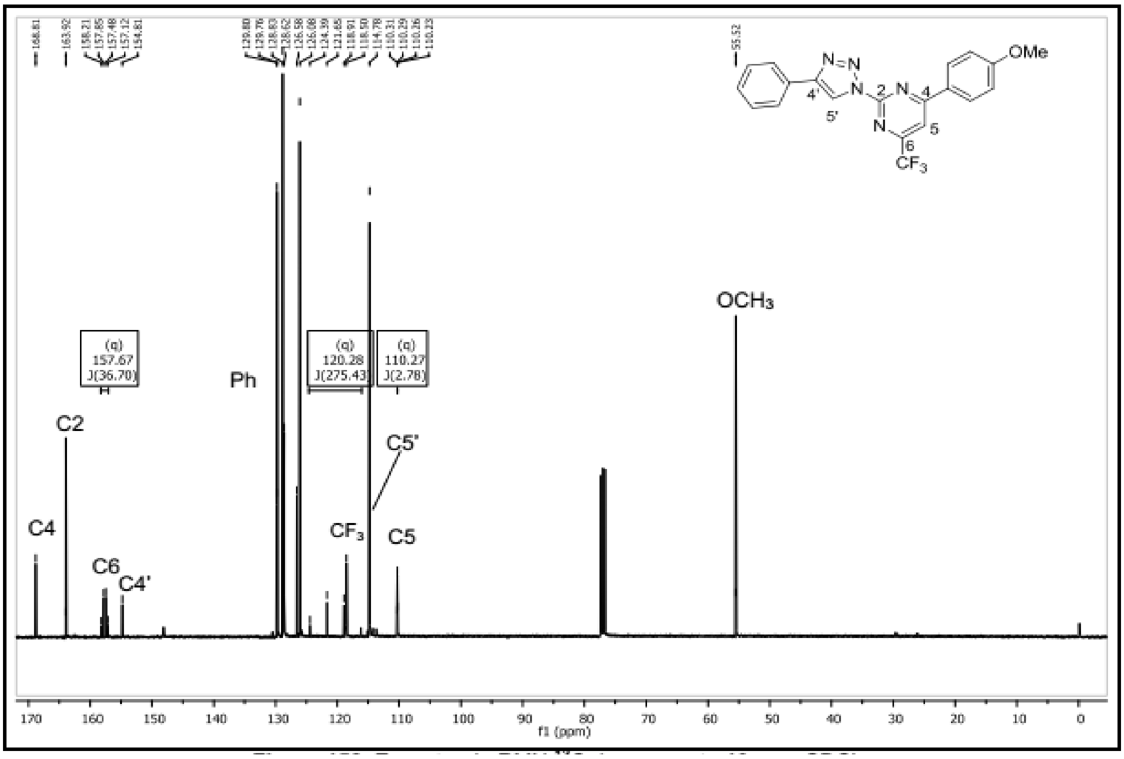

Figure S36: ^{13}C NMR of compound **8c** in $CDCl_3$.

[1] L. J. Farrugia, "ORTEP -3 for Windows - a version of ORTEP -III with a Graphical User Interface (GUI)," *J. Appl. Crystallogr.*, vol. 30, no. 5, pp. 565–565, Oct. 1997.
